# Evidence that Indo-Pacific bottlenose dolphins self-medicate with invertebrates in coral reefs

**DOI:** 10.1016/j.isci.2022.104271

**Published:** 2022-05-19

**Authors:** Gertrud E. Morlock, Angela Ziltener, Sascha Geyer, Jennifer Tersteegen, Annabel Mehl, Tamara Schreiner, Tamer Kamel, Franz Brümmer

**Affiliations:** 1 Department of Food Science, Institute of Nutritional Science, and TransMIT Center for Effect-Directed Analysis, Justus Liebig University Giessen, 35392 Giessen, Germany; 2 Department of Anthropology, University of Zurich, 8057 Zurich, Switzerland; 3 Dolphin Watch Alliance, 8057 Zurich, Switzerland; 4 Technical University Berlin, Campus El Gouna, El Gouna 84513, Egypt; 5 Research Unit for Biodiversity and Scientific Diving, Institute of Biomaterials and Biomolecular Systems, and the Scientific Diving Center, University of Stuttgart, 70569 Stuttgart, Germany; 6 Red Sea Marine Parks Authority, El Sakalla – Marina Square, Hurghada, Red Sea Governorate, Egypt

**Keywords:** Analytical chemistry, Ecology, Biological sciences, Zoology, Biochemistry

## Abstract

Indo-Pacific bottlenose dolphins (*Tursiops aduncus*) have been observed queueing up in natural environments to rub particular body parts against selected corals (*Rumphella aggregata, Sarcophyton* sp.) and sponges (*Ircinia* sp.) in the Egyptian Northern Red Sea. It was hypothesized that the presence of bioactive metabolites accounts for this selective rubbing behavior. The three invertebrates preferentially accessed by the dolphins, collected and analyzed by hyphenated high-performance thin-layer chromatography contained seventeen active metabolites, providing evidence of potential self-medication. Repeated rubbing allows these active metabolites to come into contact with the skin of the dolphins, which in turn could help them achieve skin homeostasis and be useful for prophylaxis or auxiliary treatment against microbial infections. This interdisciplinary research in behavior, separation science, and effect-directed analysis highlighted the importance of particular invertebrates in coral reefs, the urgent need to protect coral reefs for dolphins and other species, and calls for further vertebrate-invertebrate interaction studies.

## Highlights

• Dolphins rubbed body parts against specifically selected corals and sponges
• Behavioral studies were linked with hyphenated bioanalytical technique
• Vertebrate-invertebrate interaction in coral reefs may serve self-medication
• Molecular formulae were assigned to known and unknown bioactive molecules

## Graphical abstract

**Figure:**
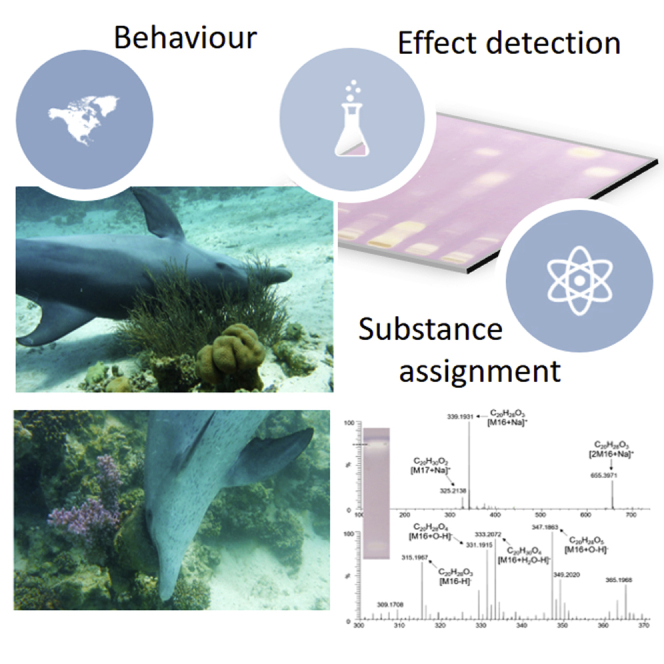

## Introduction

Rubbing behavior on distinct substrates is part of the natural physical contact behavior in cetaceans and has only been observed in a few odontocetes, for example in killer whales (*Orcinus orca*) (Ford et al., 2000) and beluga whales (*Delphinapterus leucas*) (Smith et al., 1992). The repeated rubbing behavior of wild Indo-Pacific bottlenose dolphins (*Tursiops aduncus*) around Hurghada and El Gouna in the Northern Red Sea, Egypt, against three distinct marine invertebrates is reported here. The dolphins glide toward and rub their skin against the following invertebrates accessed selectively and preferentially: the gorgonian coral *Rumphella aggregata*, the leather coral *Sarcophyton* sp., and the sponge *Ircinia* sp. They use distinct substrates for particular body parts due to the unique properties of the invertebrates (i.e. texture) and differences in the sensitivity of their body parts (i.e. strong head rubbing against the harder sponge structure). Up to now, the intention behind this rubbing behavior has been unclear. We hypothesized that this behavior serves the purpose of self-medication. Similar *Rumphella* species are known to produce antimicrobial as well as cytotoxic secondary metabolites (Sung et al., 2007b; Alarif 2012; El-Ezz et al., 2017), and also *Ircinia* sp. (Bifulco et al., 1995; Hardoim and Costa 2014; Mioso et al., 2017) and *Sarcophyton* sp. (Gomaa et al., 2016) are reported to contain bioactive metabolites. Thus, our hypothesis that dolphins utilize these secondary metabolites against dermal disease-causing pathogens (Harzen and Brunnick 1997; van Bressem et al., 2008; Kiszka et al., 2009; Hart et al., 2012) in the sense of preventative or curative self-medication (zoopharmacognosy) (Huffman 1997) appears plausible. Dolphins can suffer from viruses (van Bressem and van Waerebeek 1996; Esperón et al., 2008) and bacterial infections (Palmer et al., 1991; Russo et al., 2018); however so far, zoopharmacognosy has never been reported in any cetacean (Ansari et al., 2013). Reasons may be due to the paucity of systematic underwater observations of cetaceans, and otherwise limited on-boat surveys providing little behavioral data. Through a unique combination of boat and underwater surveys using SCUBA diving, a resident population of about 360 Indo-Pacific bottlenose dolphins in the Northern Red Sea has systematically been studied by the Dolphin Watch research team since 2009 (Kleinertz et al., 2014). So far, only the gorgonian rubbing behavior (gorgoning) has been highlighted in the film Blue Planet II Episode 1 of the BBC Natural History Unit (Attenborough 2017). Similar reports in the Bahamas (Herzing, 2015) and in Florida (Precht et al., 2018) substantiate our observations. Given that the dolphins rub particular body parts against specific invertebrates, we postulate that bioactive metabolites incorporated in the invertebrates are explicitly sought out by them for targeted self-medication.

## Results and discussion

### Rubbing behavior on selectively accessed invertebrates

**Figure 1:**
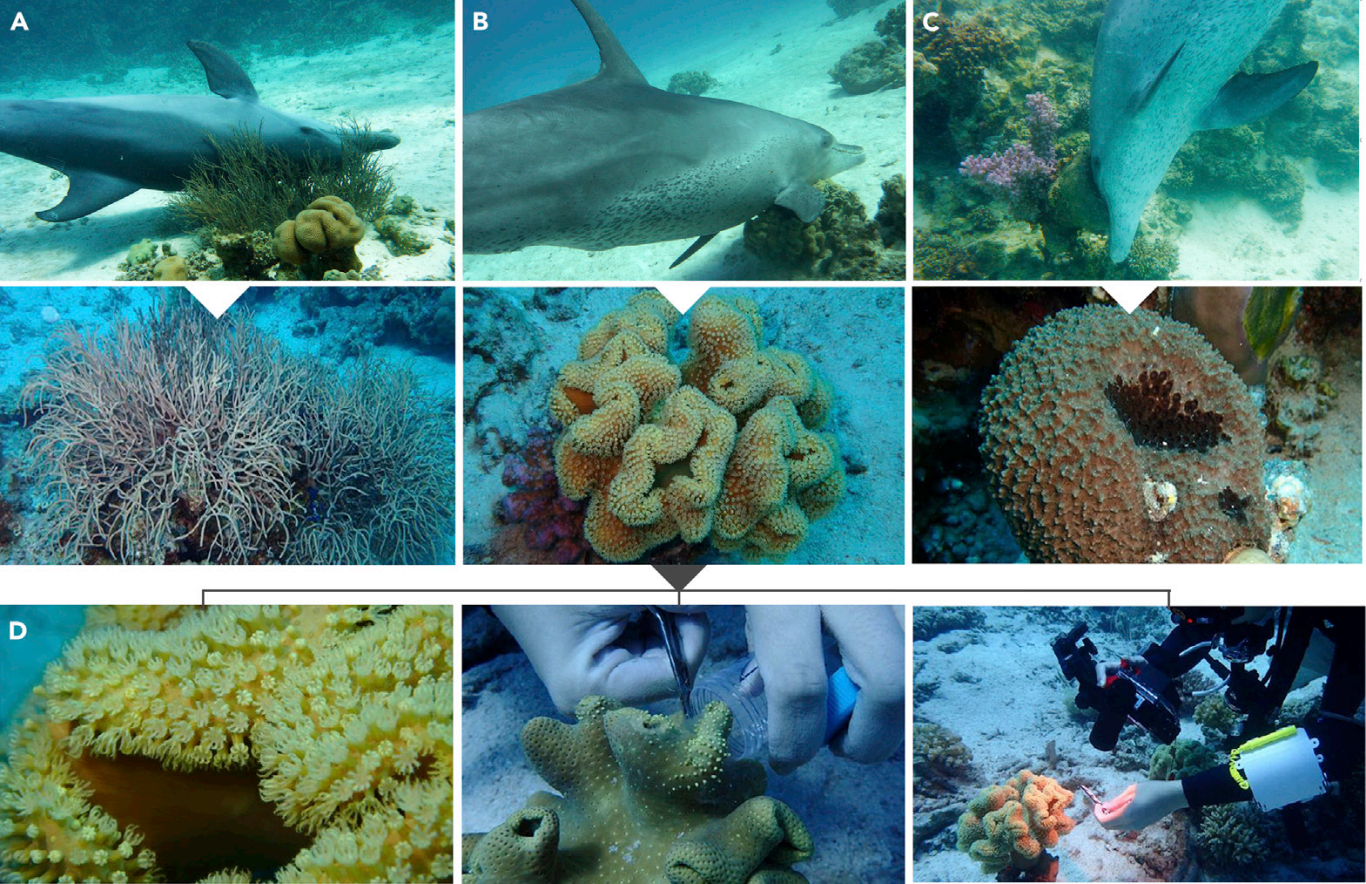
Rubbing behavior of dolphins on three specific marine organisms and underwater photo documentation of sampling Rubbing behavior on (A) gorgonian coral *Rumphella aggregata*, (B) leather coral Sarcophyton sp., and (C) sponge Ircinia sp.; (D) underwater photo documentation of sampling and location, exemplarily shown for the leather coral. Respective scale bars and further details are given in Figures S3–S5.

Indo-Pacific bottlenose dolphins around Hurghada and El Gouna in the Northern Red Sea (Figure S1) glide toward and rub their skin against the selectively accessed gorgonian coral *Rumphella aggregata*, the leather coral *Sarcophyton* sp., and the sponge *Ircinia* sp. (Table S1 Compilation of the organisms, related to Figures 1 and S1). On the gorgonian coral, dolphins slide into the branches of the coral and often repeat this behavior, so several body parts are rubbed (Videos S1 and S2 Gorgonian rubbing behavior, related to Figure 1A and S2). Upon rubbing, the gorgonian coral polyps start to secrete mucus and to close and the mucus secreted by the corals can then be transferred to the skin of the dolphin. Through the closed polyps and resultant harder and rougher surface of the coral, skin contact via abrasion and subsequent absorption might be even more efficient. Leather corals and sponges are more compact and harder in their texture than the soft gorgonian coral branches, so the dolphins push one isolated body part strongly into the substrates. For example, the dolphin rubs its ventral, lateral, or dorsal body part on the leather coral (Figure 1B). Its head and fluke often touch the coral, too or as a further example, the dolphin rubs its ventral or dorsal body part and fluke on the sponge and pushes its head strongly against it and twists it around (Figure 1C).

Video S1. Gorgonian rubbing behavior (gorgoning) Rubbing behavior of a group of Indo-Pacific bottlenose dolphins (*Tursiops aduncus*), queuing up to rub on the gorgonian coral (Rumphella aggregata) in Shaab El Erg, Red Sea, Egypt, related to Figure 1.

Video S2. Gorgonian rubbing behavior (gorgoning) Rubbing behavior of a group of Indo-Pacific bottlenose dolphins (*Tursiops aduncus*), queuing up to rub on the gorgonian coral (*Rumphella aggregata*) in Shaab El Erg, Red Sea, Egypt. At the end of the recording, a half-year-old calf watches his mother doing the gorgoning. related to Figure 1.

When in groups, dolphins are often observed queueing up behind each other to wait their turn to approach the invertebrate. This group event has been observed for the gorgonian coral and sponge, but not for the leather coral. The more sensitive calves aged under one year have not been observed engaging in the group rubbing on these particular organisms, instead they watch the adults doing the rubbing (Video S2 Gorgonian rubbing behavior, related to Figure 1A). As they start to become integrated into the group rubbing event, the calves carefully rub themselves on the substrate by touching it with certain body parts quickly (Video S3 Gorgonian rubbing behavior in group with calf, related to Figure 1A). This would imply that the behavior and any potential knowledge about the effects of the bioactive compounds contained within the invertebrates is not innate but acquired through processes of social learning (Box and Gibson 1999) and therefore socially transmitted to next generations (Krützen et al., 2005; Sargeant and Mann 2009; van Schaik and Burkart 2011). If this learned behavior serves the purpose of self-medication, these specifically accessed invertebrates must contain bioactive compounds.

Video S3. Gorgonian rubbing behavior in group with calf (gorgoning) Rubbing behavior of a group of Indo-Pacific bottlenose dolphins (*Tursiops aduncus*), queuing up to rub on the gorgonian coral (*Rumphella aggregata*) in Shaab El Fanous, Red Sea, Egypt. A one-year-old calf carefully rubs itself on the Gorgonian coral by touching it with its belly, related to Figure 1.

If dolphins rub intensively on the substrate, the substances are released (Video S4 Sponge rubbing behavior, related to Figure 1C). Sometimes dolphins will open their mouths during rubbing, making the direct contact with the invertebrate compounds obvious. An even more intense contact behavior was observed during leather coral rubbing. Dolphins sometimes extract the invertebrate from the ground and carry it in their mouths for a few minutes, the dolphin may then swing the leather coral around, causing compounds to visibly leak out of the coral and spread around the head and rostrum (Video S5 Dolphin carrying a leather coral, related to Figure 1B). These particular body parts of the dolphin can subsequently be stained a striking yellowish or greenish coloration due to the compounds excreted by the invertebrates.

Video S4. Sponge rubbing behavior Indo-pacific bottlenose dolphins (*Tursiops aduncus*) rub particular body parts on a sponge (*Ircinia sp.*) in Shaab El Fanous, Red Sea, Egypt, related to Figure 1.

Video S5. Dolphin carrying a leather coral An Indo-Pacific bottlenose dolphin (*Tursiops aduncus*) female carries a leather coral (*Sarcophyton sp.*) in her mouth for a few minutes in Shaab El Erg, Egypt. When carrying it, the dolphin often swings the leather coral around. related to Figure 1.

### Sampling of the accessed invertebrates

Interference with nature was kept to a minimum during the sampling of the substrates, and the amount of sample taken from its natural habitat was as minimal as possible. Underwater photo documentation precisely traced the natural habitats and environment around the El Gouna and Hurghada area (Figures S3−S5). The collected samples at the sites visited by the dolphins (Figure S1) were transported aboard on a mixture of ice cubes and salt and stored at −20°C in the laboratory. The metabolite profile can alter during transportation, so methanol was added immediately after the dive to one set of samples. Despite potential reaction with aldehydes to hemiacetals, methanol was preferred based on previous bioprofiling studies which allowed the most comprehensive view on active compounds present. Besides methanol, *n*-hexane was also chosen as an extraction solvent to widen the range of extractable compounds in the aplor range. The 48 extracts of the collected samples had a volume of 0.2–1.5 mL, depending on the substrate and solvent (Table S2 Sample preparation, related to STAR Methods, 0.2–1 mL for methanol extracts and 1.1–1.5 mL for *n*-hexane extracts). Such small sample volumes were challenging for analysis aimed at the detection of bioactive compounds.

### Outline of the bioanalytical screening

**Figure 2:**
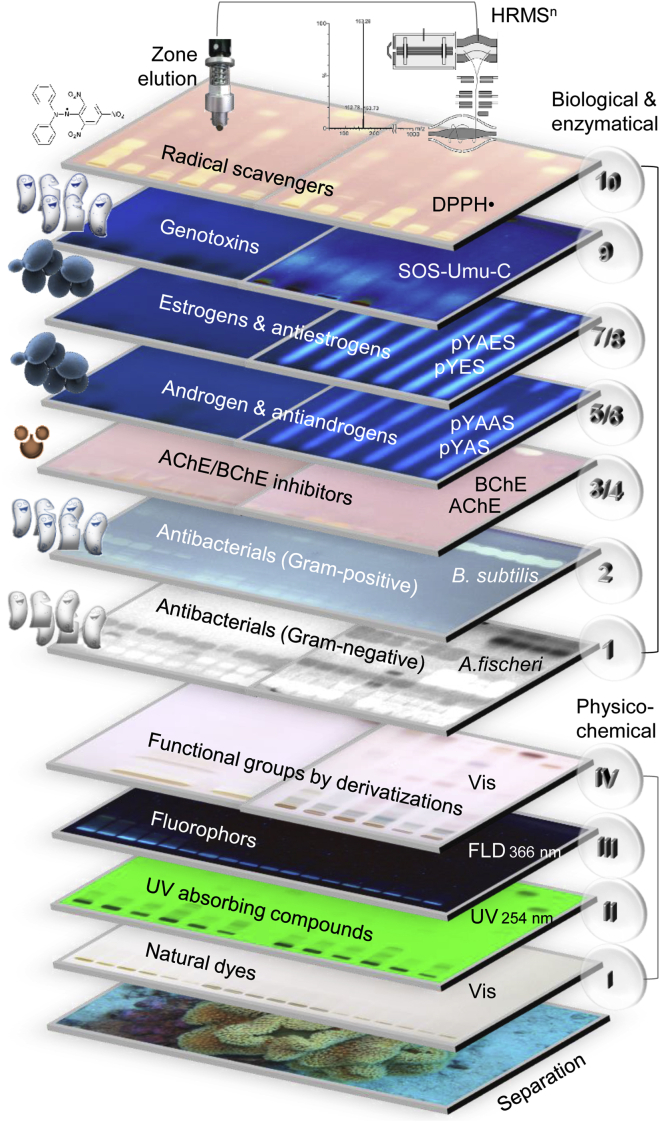
Scheme of effect-directed profiling Planar chromatographic separation of substrates was combined with (I–IV) physicochemical multi-detection at Vis/UV/FLD including chemical derivatizations, (1–10) ten different effect-directed on-surface assays (abbreviations in supplemental information), and HRMS.

**Figure 3:**
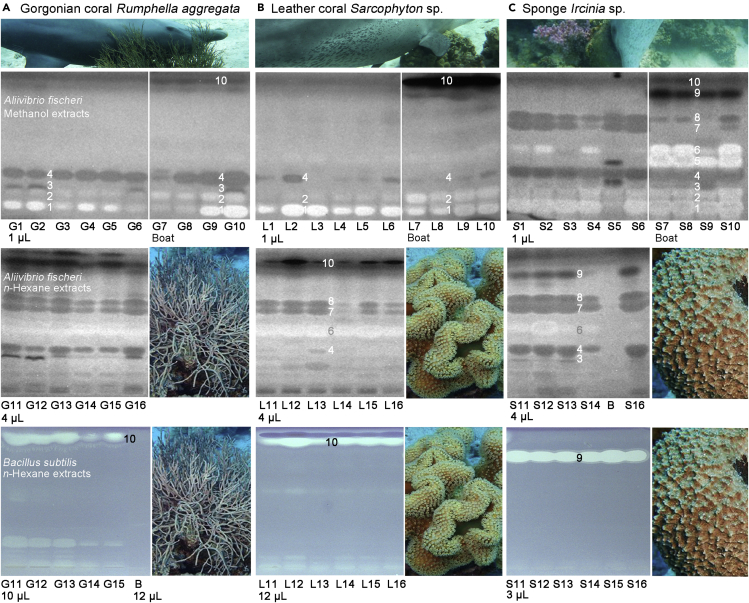
Antibacterial compounds against Gram-negative as well as Gram-positive bacteria Bioprofiling of the methanol and *n*-hexane extracts of the (A−C) three distinct substrates revealed the antibacterial compound zones 1–10 (evident as dark or bright bands), developed on HPTLC plates silica gel 60 with ethyl acetate – methanol – water 15:3:1 (*V*/*V*/*V*) and detected via the bioluminescence (bioautogram after 27 min depicted as grayscale image) of the applied Gram-negative *A. fischeri* and at white light illumination after the applied planar Gram-positive *B. subtilis* bioassay (B: solvent blank; respective pre-assay FLD 366 nm chromatograms in Figure S7).

**Figure 4:**
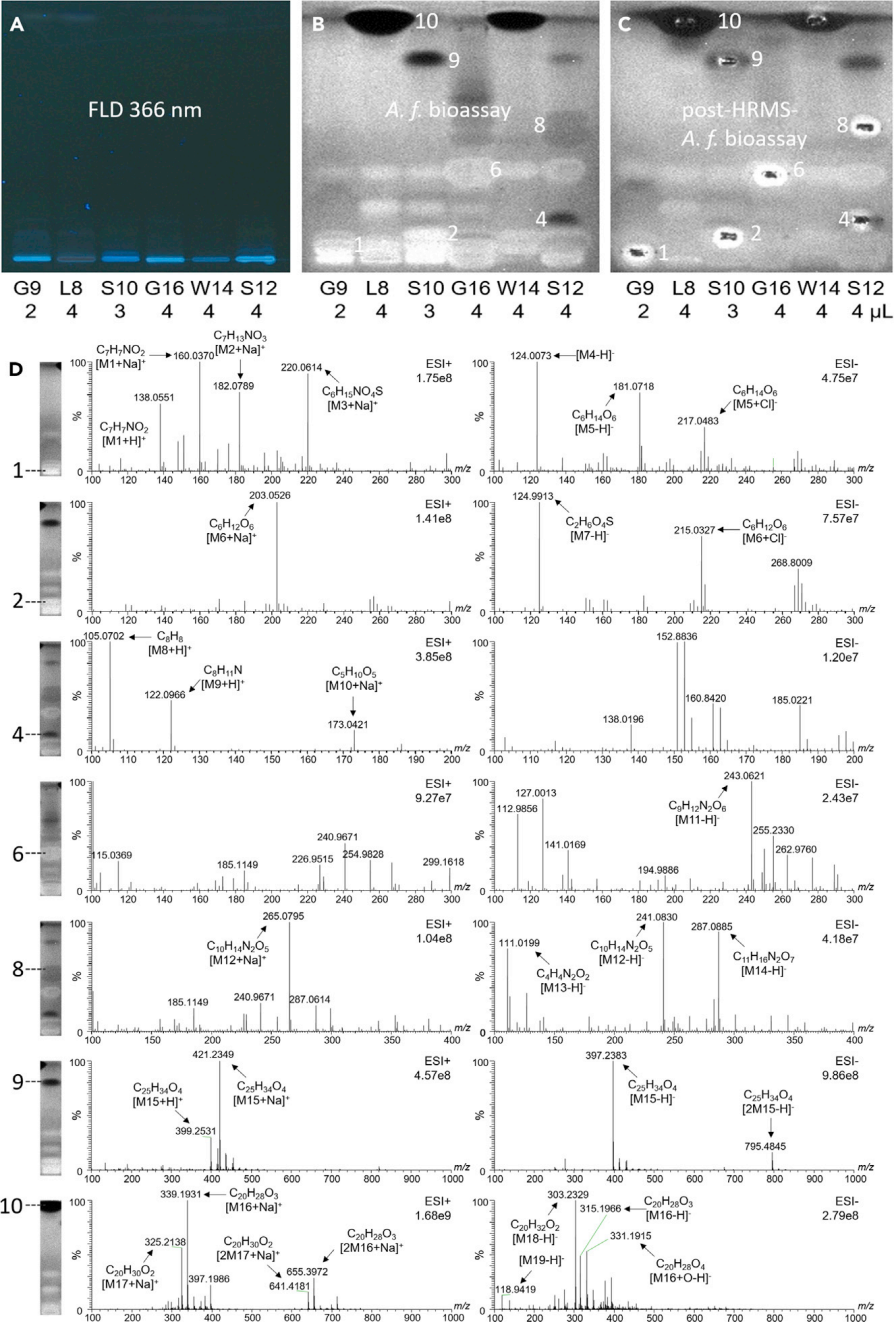
Characterization of antibacterial zones 1–10 by HPTLC-HESI-HRMS-bioassay-luminescence detection (A) Chromatogram at FLD 366 nm on HPTLC plate silica gel 60 with ethyl acetate – methanol – water 15:3:1 (*V*/*V*/*V*) of the methanol boat and *n*-hexane extracts of the three distinct substrates, (B) respective *A. fischeri* bioautogram (bioluminescence depicted as grayscale image), and (C) duplicate plate used for recording of mass spectra of zones 1–10, followed by post-HRMS *A. fischeri* bioassay application to verify the proper positioning of the elution head zones. (D) Tentative assignment of molecular formulae.

One of the main advantages of the high-performance thin-layer chromatography (HPTLC) method used (Figure S6) is the efficient use of costly samples (Klöppel et al. 2008, 2013; Mahran et al., 2019). Not even a microliter of the limited sample volume was lost during instrumental operations. As compound isolation was skipped, the direct analysis avoided any artifacts (Czarkie et al., 1985). Another advantage of the HPTLC method is the multi-detection and subsequent on-surface application of assays (Morlock 2021), if compared to status quo procedures (Kongstad et al., 2015). A mobile phase system was developed that spreads the compounds present in the extracts along the migration distance (data not shown). Ten effect-directed assays of different mechanisms (i.e., seven biological assays, two enzymatic assays, and a radical scavenging assay, Figure 2) were exploited to figure out which of the on-surface-separated metabolites had bioactive properties due to their interaction with the applied microorganism or enzyme. Depending on the assay, the generated specific response was based on color formation, fluorescence generation, fluorescence reduction (for antagonistic effects), or change in the intrinsic bioluminescence, which indicated the respective bioactive properties of a substance (Table S3 Ten different assays applied, related to Figure 2). All assays were repeated, and responses were confirmed several times. The combination of the parallel sample separation with the assay response led to expressive effect-profiles (Video S6 Bioanalytical workflow, related to Figure 2). As an alternative instrumental system, the miniaturized open source OCLab3 system (https://github.com/OfficeChromatography/OCLab3), which comprises as 2LabsToGo system both the chemistry and biology laboratory, can be used (Morlock, 2021). The subsequent targeted recording of heated electrospray ionization−high-resolution mass spectra (HESI−HRMS) of bioactive zones allowed to assign molecular formulae. Note that a fully automated workflow was reported recently (Mehl et al., 2021), which makes this hyphenation highly convenient (Video S7 Hyphenation to MS, related to Figures 2–4).

Video S6. Bioanalytical workflow Exemplarily for the planar *A. fischeri* bioassay, the step-automated workflow is depicted showing application, development (normal phase separation), piezoelectric spraying of the *A. fischeri* bioassay suspension and bioluminescence detection, related to Figure 2.

Video S7. Hyphenation to MS Fully automated on-surface autosampling of bioactive zones for liquid chromatography−mass spectrometry recording, related to Figures 2–4.

### Effect-directed profiling

After centrifugation, the supernatants of the 48 extracts were applied (1–10 μL/band). The higher methanol volume added to the boat samples was compensated by a respective higher application volume, so that the sample amount was equivalent to the respective laboratory sample in the HPTLC image. Bioactive compounds were detected in both extraction polarities in all substrates. Both extractions were complementary, as evident via the antibacterial metabolite patterns of the *Aliivibrio fischeri* bioautograms (Figure 3). The methanol extracts contained more polar bioactive compounds (*hR*_F_ < 20), which were lost in the *n*-hexane extracts, which in turn contained the middle to apolar bioactive compounds. The metabolite pattern of the methanol extracts obtained on board differed to a certain extent from that obtained in the laboratory. The differences were minor for the gorgonian corals, moderate for the leather coral, and highest for the sponge extracts. This shift in the individual intensities of bioactive compounds within a substrate can be explained by the lability of metabolites (e.g., due to oxidation) during boat transportation. Across all assays performed, the boat-methanol and *n*-hexane extracts showed comparatively stronger responses than the laboratory-methanol extracts.

The two bacterial assays were selected for the antibacterial effect detection to represent the two different bacterial cell wall types (Figure 3). The Gram-negative *Aliivibrio fischeri* bioassay was used as a starter assay, as it immediately indicates most bioactive compounds based on our experience. A rough indication of the required sample volumes to be applied was obtained as well as information on the quality of the separation regarding the distribution of bioactive zones along the separated sample track. In the first *A. fischeri* bioautogram, up to 10 different bioactive zones (zones **1**–**10**) were observed. On the one hand, up to six compounds (evident as dark bands) reduced the native bioluminescence of the bacteria, which is related to the decrease of the energetic cell metabolism and of the vitality of the bacteria. On the other hand, up to four compounds, evident as bright bands, increased the bioluminescence related to an improved energetic cell metabolism. The comparison between boat and laboratory extracts revealed only slight differences for the gorgonian corals. However, for the leather coral, the more polar bioactive compound zone **2** (*hR*_F_ 20) and the very strong lipophilic bioactive compound zone **10** (*hR*_F_ > 90) were more pronounced in the boat-methanol extracts. The difference was highest for the sponge extracts, for which the bioactive compound zones **5** and **6** (*hR*_F_ 40–50) as well as **9** and **10** (*hR*_F_ > 90) were more dominant in the boat-methanol extracts, whereas zones **7** and **8** were less intense in their response.

The second bioassay detected antibacterial compounds against Gram-positive *Bacillus subtilis* bacteria. The antibacterial responses were more pronouncedly detectable in the *n*-hexane than methanol extracts. The applied volumes were adjusted depending on the three distinct substrates. Approximately at the same upper position as in the previous bioassay, the two apolar antibacterial zones **9** and **10** were evident as colorless (white) bands in the bioautogram of the *n*-hexane extracts of the three distinct substrates (Figure 3). The latter two zones were strongest in their response and prominently acted against both bacterial cell wall types, whereby zone **9** was predominantly present in the sponges and zone **10** in the corals. Because in the first two bioassays and in the multi-detection imaging, the intra-species responses were similar, and due to the limited volume of sample extracts, further analyses were not performed with all but only with selected representatives. These were also replaced during assay repetitions and resulted in the same responses, confirming that similar results were obtained within a species.

The third and fourth enzymatic assays detected inhibitors of the acetyl- and butyrylcholinesterases (AChE/BChE). Activity responses were more pronounced in the *n*-hexane and boat-methanol extracts than in the methanol extracts (Figures S8, S9A and S9B). At the same upper position as in the previous two bioassays, the two zones, zones **9** and **10**, were observed as colorless (white) inhibition bands. Only the leather corals revealed a pronounced response for zone **10** in both inhibition assays. The much weaker inhibiting zone **9** was only observed in the sponge extracts. The fifth planar yeast estrogen screen (pYES) bioassay detected the blue fluorescent estrogen-like compound zones **11**–**13**, mainly in the methanol boat extract of the leather coral L8 (Figures S9C). A much weaker estrogenic response was observed in the *n*-hexane extract of the gorgonian coral G11 (zone **13**) or leather coral L13 (zone **12**). In the sixth planar yeast antiestrogenic screen (pYAES) bioassay, the compound zones **14** and **15** were observed in the boat-methanol (Figure S9D, L8) and *n*-hexane extracts (L13) of the leather corals only. These zones were evident through fluorescence reduction of the estrogen 17β-estradiol, which was applied as a vertical rectangle along each separated sample track. The seventh planar yeast androgen screen (pYAS) bioassay detected androgen-like compounds as blue fluorescent zones. However, no androgen was observed for the given amounts applied (up to 10 μg/band, Figure S9E). The eighth planar yeast antiandrogen screen (pYAAS) bioassay revealed two pronounced antiandrogenic zones, as evident in the fluorescence reduction of the androgen testosterone which was applied as a vertical rectangle along each sample track. These antiandrogenic responses were at the same *hR*_F_ values as the previously detected antiestrogenic compound zones **14** and **15** (Figure S9F, L8/L13), again observed only in the leather corals. The ninth genotoxicity (SOS-Umu-C) bioassay using *Salmonella typhimurium* bacteria was performed not only on the normal phase but also on the reversed phase plate. The application of both allowed a complementary perspective on the samples. The more apolar genotoxic compound zone **16** was only detected in the leather corals (Figure S9G, L8/L13) on the normal phase plate. This normal phase system detects the zones more sensitive, and hence, this weak genotoxic zone was not observed in the reversed phase system. On the reversed phase plate (Figure S9H), a further weak genotoxic response (zone **17**) was observed for all tested substrates. Although the RP-HPTLC system leads to much sharper zones than the NP-HPTLC system for long incubation times in polar bioassay media (Klingelhöfer and Morlock 2014), this already diffused apolar zone is even more prone to diffusion on the normal phase bioassay plate, on which it was thus not detectable. The tenth 2,2-diphenyl-1-picrylhydrazyl (DPPH^⋅^) assay revealed up to six different radical scavenging (antioxidant) compound zones (Figure S10A). Among these, the previously discovered more apolar bioactive zones **9** and **10** in the leather coral and sponge extracts, respectively, stand out, whereas the gorgonian coral extracts contained several intense polar antioxidants.

Across the ten assays and the multi-imaging performed (Figures 2 and 3, and S7–S11), the obtained effect-profiles of the 48 extracts were similar between species or within a phylum, as evident for the gorgonian coral and leather coral. They differed more between different phyla (corals *versus* sponge). Bridging the gap between chromatography and toxicology, the effect-directed profiles provided information on biological activity. Such responses could be explained by the developed antimicrobial defense strategies of the sessile marine microorganisms, but it can also contribute to a certain extent to the homeostasis of the skin of the dolphins in case of parasite infestation. The bioactive metabolites discovered in the three substrates, which were exclusively accessed by the dolphins, can explain the purpose of self-medication by rubbing on the substrates. These metabolites can come into contact with the skin of the dolphins and could be useful for skin protection or as an auxiliary treatment against bacterial infections or for balancing the hormonal status or homeostasis of the skin.

### Physicochemical profiles of the sampled substrates

Additional information on spectral characteristics and on polarity of the compound zones was obtained by chemical derivatization reactions. For example, the FLD 366 nm chromatograms recorded before application of the assays (Figures S7 and S8) showed the start zone as the main fluorescent zone. The Vis chromatogram did not show any visible responses (not shown). However, the physicochemical detection of the derivatized chromatograms revealed the bioactive zones **9** and **10** after derivatization with the diethylamine aniline sulphuric acid reagent (used for detection of glycosides or lactones, Figure S10B) and with the vanillin sulfuric acid reagent (used for a more universal detection of organic compounds, Figure S10C). The natural product reagent for detection of molecules with adjacent keto/hydroxy groups did not show any response (not shown), whereas both zones were detected by the primuline reagent, which points to lipophilic compounds (Figure S11). Both zones did also absorb at UV 254 nm (Figure S11). Although dedicated structure elucidation experiments are needed to identify molecular structures, the here obtained information can confirm tentative assignments. For example, zones **9** and **10** (*hR*_F_ > 90) were not natively visible or fluorescent, but UV-active (Figure S11). Both were assigned a glycosidic or lactonic (Figure S10) and lipophilic (Figure S11) structural moiety, as proven by the use of derivatization reagents. Both zones showed a pronounced antibacterial (Figure 3) and radical scavenging (antioxidative) activity (Figure S10A). Zone **10** was a comparatively stronger inhibitor of AChE and BChE than zone **9** (Figures S9A and S9B). Based on these data, it was hypothesized that both zones were a lipolactone due to their high *hR*_F_ value in the given chromatographic system, UV-activity, and responses in both antibacterial bioassays as well as in the radical scavenging assay. Antibacterial and antioxidative properties of lactones are known (Mar’in et al., 2002; Shoaib et al., 2017; Zhang et al., 2020).

### Characterization of antibacterial zones 1–10 by HPTLC−HESI-HRMS−bioassay−bioluminescence

**Figure 5:**
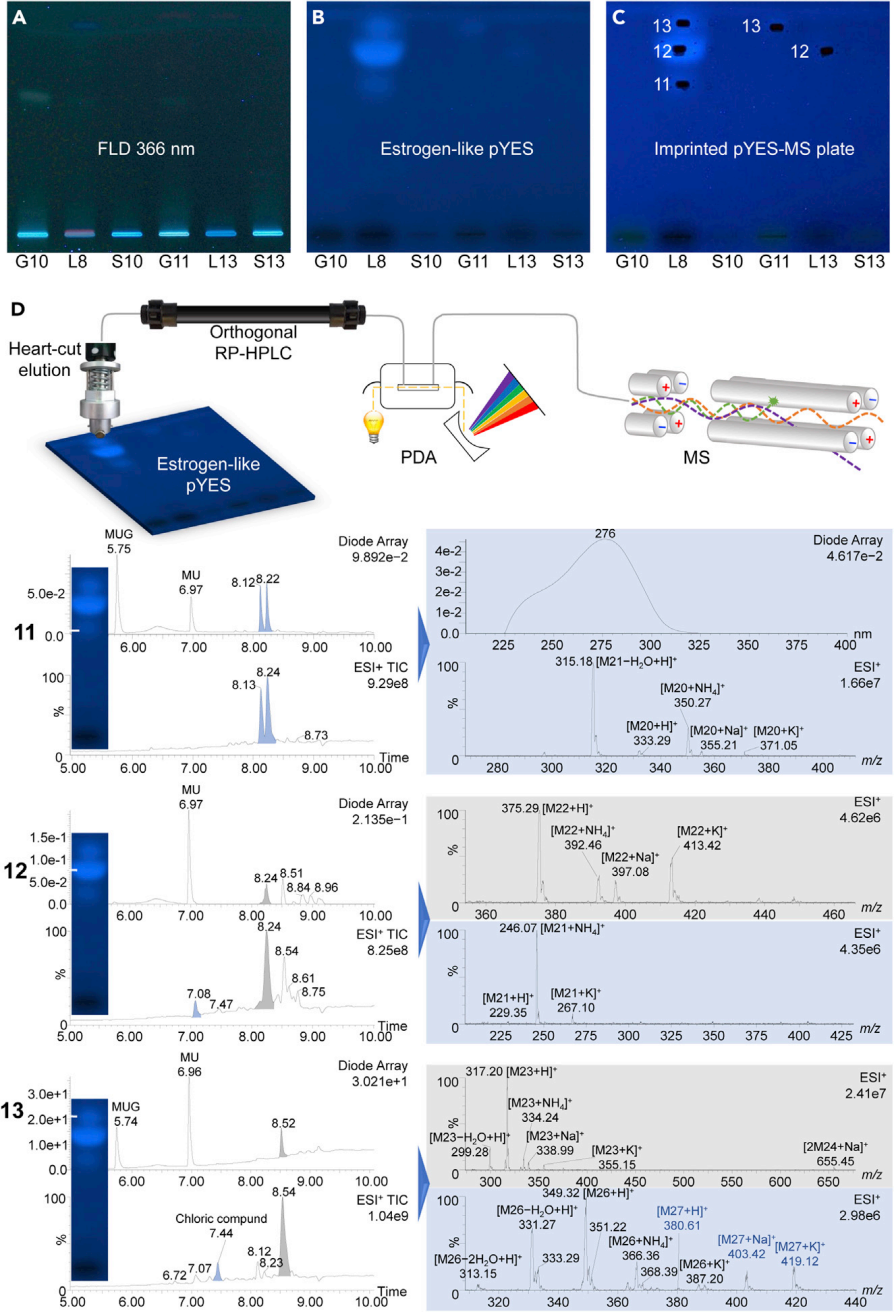
Characterization of estrogenic zones 11–13 directly out of the pYES bioautogram (A) Chromatogram at FLD 366 nm on HPTLC plate silica gel 60 with ethyl acetate – methanol – water 15:3:1 (*V*/*V*/*V*) of the extracts of the three distinct substrates, (B) pYES bioautogram, and (C) elution head imprint, which verifies the proper positioning of the elution head on the zones. (D) Scheme of the zone characterization by NP-HPTLC-pYES bioassay-RP-HPLC-DAD-HESI-MS showing recorded PDA, MS-TIC chromatograms and extracted mass spectra (same colour) with assigned mass signals.

The *A. fischeri* bioluminescence enhancing or decreasing zones **1**–**10** (Figure 3) were further characterized by online elution to HESI-HRMS. The plate was prepared twice. One plate was directly subjected to the bioassay used for zone marking on the other plate. The latter plate was used for recording of HRMS spectra and subsequent bioassay application to prove the proper positioning and zone transfer by the match of the stamped oval elution head imprint with the post-HRMS bioautogram (Figure 4A–4C). The targeted recording of HRMS spectra led to pronounced mass-over-charge signals (*m*/*z*) and respective molecular formulae were assigned after a detailed literature study (Table S4 Compilation of activities related to Figure 4, and Table S5 Assignment of the mass signals, related to Figure 5). The formation of multiple adducts allowed the multiple confirmation of the assigned molecular formulae, and is explained by the zone elution from the bioautogram containing salt-rich bioassay media. Recently, it was reported that inorganic sulfur can be assimilated from the seawater by corals (Higuchi et al., 2021) even in the absence of symbionts (Erwin et al., 2012). Apart from natural halogenated products, also xenogenic chlorinated molecules were found in corals (van der Schyff et al., 2021). Both references support the assignment of the sulfur- or chlorine-containing molecular formulae obtained (Table S5 Assignment of the mass signals, related to Figure 4).

In accordance with literature (Table S5 Assignment of the mass signals, related to Figure 4), the HRMS signals of zone **9** (basepeak 397.2383 [M15–H]^–^, C_25_H_34_O_4_, Δppm 0.03) in the sponge were tentatively assigned to the structural isomers fasciculatin/variabilin/palinurin (Ishibashi et al., 1993), all furanosesterterpene tetronic acids and thus lipolactones. Fasciculatin has been isolated from *Ircinia fasciculata* (Cafieri et al., 1972), present in digestive glands, mucus, and mantle sections (Marin et al., 1997), in the Thyrrenean Sea (Rosa et al., 1997) and Atlantic Coast of Morocco reporting its moderate cytotoxicity as defense strategy (Rifai et al., 2005). Variabilin has been isolated from *Ircinia variabilis* showing antibiotic activity (Faulkner 1973), also against Gram-positive *Sarcina lutea* (minimum inhibitory concentration of 66 pg/mL). Palinurin has been isolated from *Ircinia variabilis* (Alfano et al., 1979) and was proven to be mainly responsible for its antimicrobial activity (Martí et al., 2003). These reports are in full agreement with the strong effects against Gram-positive and Gram-negative bacteria of zone **9** in the sponge extracts in the *A. fischeri* bioautogram (Figures 3 and 4). The HRMS signals of zone **10** (basepeak *m/z* 339.1931 [M16 + Na]^+^, C_20_H_28_O_3_, Δppm 0.2) in the leather corals were tentatively assigned to the isomers sarcophine/sarcophytolide/sarcophytolide B or C (Figures 3, 4 and S12). Sarcophytolide was reported to act as an antimicrobial (Gomaa et al., 2016), which corresponded to our results. Though isomers were not distinguished, both tentative zone assignments were considered as proof of the bioprofiling. The mass signal (basepeak 203.0526, [M6+Na]^+^, C_6_H_12_O_6_) obtained from the bioluminescence enhancing zone **2** (more or less present in the three substrates) pointed to glucose. This finding is in agreement with the rise in blood glucose levels after intake of sponge extracts (Ajabnoor et al., 1991), or the glucose uptake by corals (Stephens 1960). The obtained mass signal (C_6_H_14_O_6_, basepeak 181.0718 [M5–H]^–^) of the bioluminescence enhancing zone **1** can be explained by reduction of glucose to a sugar alcohol (hexane-hexol).

### Characterization of hormone-like zones 11–15 by NP-HPTLC−bioassay−RP-HPLC−PDA−HESI-MS

A recently developed 8D hyphenation (Schreiner and Morlock 2021) was alternatively used to further characterize the hormonal-effective compound zones. This hyphenation allowed eluting the bioactive zone from the bioautogram into the second separation and photodiode array (PDA) detection dimension (RP-HPLC-PDA-HESI-MS). By the fully automated workflow, hormonal-effective compound zones were eluted directly out of the highly salted bioautogram, which still contained the genetically modified yeast cells with the human estrogen/androgen receptor incorporated. The zone eluate was automatedly transferred via online desalting to RP-HPLC−PDA−HESI-MS (Figures 5 and S13). The connection to the HRMS system (to assign molecular formulae) would have been the best choice, but was not available. Predominantly in the leather corals, zone **12** was strongest in the estrogen-like response, whereas zones **11** and **13** were comparatively weaker (Figures 5B and S9C). Although less intense than in the leather coral L8, zone **12** was also detected in the leather coral L13, and zone **13** in the gorgonian coral G11. Androgenic compounds were not detected for the amounts applied (up to 10 μg/band, Figures S9E and S13B). The elution head was properly positioned on the zones, as proven by the remaining oval elution head imprint (Figures 5B and 5C).

The RP-HPLC−PDA−HESI-MS data were obtained (Figure 5D). The substrate MUG and the fluorescent cleavage product MU were also observed and assigned in the RP-HPLC−PDA chromatogram. Zone **11** (Figure 5D) was separated into two peaks at RT 8.13 and 8.24 min, which were proven to be isomers based on the same mass signals observed. Both isomers showed a maximal absorbance at 276 nm. The mass spectral data obtained by RP-HPLC−PDA−HESI-MS revealed multiple adduct formations (Table S6 Assignment of the mass signals, related to Figure 5), all belonging to the same neutral molecule of 332 Da. Zone **12** (Figure 5D) was separated into two peaks at RT 7.08 and 8.24 min. The observed mass signals for the first peak at RT 7.08 min showed multiple adduct formations (Table S6 Assignment of the mass signals, related to Figure 5), which evidenced a neutral molecule of 228 Da. In the planar chromatogram, neither a UV/Vis absorbance nor fluorescence at 366 nm was detected. This lack in a chromophore was confirmed by the following photodiode array detection (Figures 5A and 5D). The mass spectral data of the second peak at RT 8.24 min revealed multiple adduct formations (Table S6 Assignment of the mass signals, related to Figure 5), which evidenced the neutral molecule of 374 Da (a different molecule was clearly obtained despite the same retention time as for zone 11).

The estrogen-like zone **13** (Figure 5D) and the antiandrogenic zone **14** (Figure S13D), both located at the same *hR*_F_ value on the pYES and pYAAS bioautograms, respectively, showed the same two peaks with the same spectral data. The first peak at RT 7.44 min contained two different compounds, which pointed to the neutral molecules of 348 Da and 380 Da based on their pronounced multiple adduct formations (Table S6 Assignment of the mass signals, related to Figure 5). The molecule of 348 Da showed a distinct chlorine isotope pattern and clearly contained one chlorine atom in the molecule (Figure 5D). The second peak at RT 8.54 min evidenced the neutral molecule of 316 Da, which was proven by the multiple adduct formations (Table S6 Assignment of the mass signals, related to Figure 5). The same mass signals indicating the neutral molecule of 316 Da were determined in the antiestrogenic/antiandrogenic zone **15** (Figure S13D), which was considered to be a structural isomer of zone **14**. A further peak at RT 7.09 min pointed to the positively charged molecule of 366 Da, evidenced by its multiple adducts (Table S6 Assignment of the mass signals, related to Figure 5). Note that the clarification of the effect-responsible HPLC peak needs an individual collection after the PDA detection, followed by the HPTLC−bioassay. The MS recording of bioactive compound zones directly out of the bioautogram was found to be advantageous, as all signals obtained were confirmed by the multiple adduct formations (caused by the assay salts present). This corroborated the assignment of each mass signal.

### Limitations of the study

This interdisciplinary study provides evidence of self-medication where dolphins rub on specific invertebrates in the natural environment and shows the relationship between this behavior and the presence of bioactive metabolites. Meaningful effect profiles were obtained by linking behavioral studies with a hyphenated bioanalytical technique, performed at a minimally exploitative environmental level. We combined the disciplines of biology, biochemistry, separation science, and high-resolution mass spectrometry on a single adsorbent surface to collect information on the activity of compounds and to identify them. Sampling was performed according to Egyptian regulations, and a major challenge for the bioanalytical procedure was the limited small sample volume of only 0.2–1.5 mL per sample. From such small sample volumes, ten effect-directed assays of different mechanisms and then the characterization of the most important bioactive compounds had to be performed. To the best of our knowledge, any other technique would have required a much larger sample volume to achieve the same results obtained. The 17 bioactive compounds detected in the three invertebrates selectively accessed by the dolphins showed antibacterial (against Gram-positive and Gram-negative bacteria), antioxidative, acetyl-/butyrylcholinesterase inhibiting, estrogenic, antiestrogenic, antiandrogenic, and genotoxic activities. In a straightforward way, molecular formulae were obtained from these bioactive zones. The identification of some molecules that were already known (such as isosarcophytoxide, sarcophytol B, and the isomers of fasciculatin/variabilin/palinurin and sarcophine/sarcophytolide/sarcophytolide B/sarcophytolide C) was considered as proof of principle for properly detecting further unknown ones, which could not be assigned to substances described in the literature and still need to be identified or structure elucidated. However, the latter would require the collection of much larger quantities of invertebrate samples. Nevertheless, the detected effects and assignments in turn have provided an explanation (medication purpose) for the astonishing rubbing behavior of the dolphins. Ultimately, this study shows the relationship between the selective rubbing behavior of Indo-Pacific bottlenose dolphins and the presence of bioactive substances, which may provide evidence for their self-medication. Moreover, it calls for further research on vertebrate-invertebrate interaction in coral reefs, and draws attention to the social intelligence of dolphins, the need for interdisciplinary and hyphenated bioanalytical thinking, and to the importance of efforts to conserve this important habitat for marine life.

## Data availability

All data are available in the main text or supplemental information. The datasets generated during the study are available from the corresponding author on reasonable request. New codes were not created.

## Ethical statement

We are committed to climate change harm prevention, integrity of scientific data and evidence, solidarity, gender balance, sustainability, justice and equity, and a precautionary attitude.

## STAR★Methods

### Key resource table

**Table:** 

REAGENT or RESOURCE	SOURCE	IDENTIFIER
**Biological samples**		

*Rumphella aggregata*	Shaab El Erg and Shaab El Fanous, Northern Red Sea, Egypt	Red Sea Marine Parks Authority, El Sakalla – Marina Square, Hurghada, Red Sea Governorate, Egypt
*Sarcophyton* sp.	Shaab El Erg and Shaab El Fanous, Northern Red Sea, Egypt	Red Sea Marine Parks Authority, El Sakalla – Marina Square, Hurghada, Red Sea Governorate, Egypt
*Ircinia* sp.	Shaab El Erg and Shaab El Fanous, Northern Red Sea, Egypt	Red Sea Marine Parks Authority, El Sakalla – Marina Square, Hurghada, Red Sea Governorate, Egypt

**Chemicals, peptides, and recombinant proteins**		

Methanol, 99.9%	Merck, Darmstadt, Germany	CAS No. 67-56-1
*n*-Hexane, 95%	Tedia Company, Fairfield, OH, USA	CAS No. 110-54-3
Methanol, chromatography grade	MS-grade, Honeywell, Morristown, NJ, USA	CAS No. 67-56-1
Acetone, chromatography grade	Merck, Darmstadt, Germany	CAS No. 67-64-1
Ethyl acetate, chromatography grade	Sigma–Aldrich–Fluka, Steinheim, Germany	CAS No. 141-78-6
Müller–Hinton broth, for microbiology	Sigma–Aldrich–Fluka, Steinheim, Germany	www.sigmaaldrich.com/DE/en/product/sial/70192
Lysogeny broth (LB, Lennox) powder, including 5 g/L sodium chloride	Sigma–Aldrich–Fluka, Steinheim, Germany	www.sigmaaldrich.com/DE/en/product/sigma/l3522
Dimethyl sulfoxide (DMSO), ≥99.8%	Carl Roth,Karlsruhe, Germany	CAS No. 67-68-5
3-[4,5-Dimethylthiazol-2-yl]-2,5-diphenyltetrazolium bromide (MTT), ≥98%	Carl Roth,Karlsruhe, Germany	CAS No. 298-93-1
Tris-HCl buffer, for molecular biology	Sigma–Aldrich–Fluka, Steinheim, Germany	CAS No. 1185-53-1
Citrate phosphate buffer: 6 g/L citric acid monohydrate and 10 g/L disodium hydrogen phosphate in double-distilled water, adjusted to pH 12 by solid sodium hydroxide	Sigma–Aldrich–Fluka, Steinheim, Germany	Morlock and Klingelhöfer (2014)
Diphenyl-1-picrylhydrazyl (DPPH⋅), 95%	Alfa Aesar, Schwerte, Germany	CAS No. 1898-66-4
α–Naphthyl acetate, ≥98%	Sigma–Aldrich–Fluka, Steinheim, Germany	CAS No. 830-81-9
Fast Blue B salt, 95%	MP Biomedicals, Eschwege, Germany	CAS No. 14263-94-6
Caffeine, 99%	Sigma–Aldrich–Fluka, Steinheim, Germany	CAS No. 58-08-2
Rivastigmine tartrate, 98%	Sigma–Aldrich–Fluka, Steinheim, Germany	CAS No.123441-03-2
Testosterone, ≥99%	Sigma–Aldrich–Fluka, Steinheim, Germany	CAS No. 58-22-0
Gallic acid, ≥98%	Carl Roth, Karlsruhe, Germany	CAS No. 149-91-7
Tetracycline hydrochloride, reagent grade	Serva Electrophoresis, Heidelberg, Germany	CAS No. 60-54-8
17β-Estradiol (E2), 98.5%	Dr. Ehrenstorfer, Augsburg, Germany	CAS No. 50-28-2
Acetylcholinesterase (AChE) from *Electrophorus electricus*, ≥245 U/mg, 10 kU/vial	Sigma–Aldrich–Fluka, Steinheim, Germany	CAS No. 9000-81-1
Butyrylcholinesterase (BChE) from equine serum, ≥140 U/mg	SERVA, Heidelberg, Germany.	CAS No. 9001-08-5
*Aliivibrio fischeri* NRRL-B11177	Leibniz Institute, DSMZ, Braunschweig, Germany	Strain DSM-7151
*Bacillus subtilis* subsp. s*pizizenii*	Merck, Darmstadt, Germany	Strain DSM-618

**Recombinant DNA**		

*Saccharomyces cerevisiae* BJ1991, containing the hAR expression plasmid	Xenometrix, Allschwil, Switzerland	www.xenometrix.ch/shop/XenoScreen-YAS-Strain-Screening-for-Endocrine-Disruptive-Chemical-with-Androgenic-Activity
*Saccharomyces cerevisiae* BJ3505, containing the hER expression plasmid	McDonnell et al. (1991)	https://doi.org/10.1016/0960-0760(9190038-7)
PTM™ *Salmonella typhimurium* TA1535/pSK1002	Trinova Biochem, Giessen, Germany	https://trinova.de/components_for_assay_kits.php

**Software and algorithms**		

FreeMode option of winCATS software	CAMAG, Muttenz, Switzerland	version 1.4.7.2018
VisionCATS software	CAMAG, Muttenz, Switzerland	version 3.1.21109.3
Xcalibur 3.0.63 with Foundation 3.0 SP2 software	Thermo Fisher Scientific, Bellefonte, PA, USA	version 3.0.63

Other		

Scientific diving, SCUBA diving	Confédération Mondiale des Activités Subaquatiques (CMAS) standards	www.cmas.org
*Aliivibrio fischeri* medium	Klöppel et al. (2008)	https://doi.org/10.1556/jpc.21.2008.6.7
*Bacillus subtilis* medium and substrate	Jamshidi-Aidji and Morlock (2016)	https://doi.org/10.1021/acs.analchem.6b02648
*Salmonella typhimurium* medium and substrate	Meyer et al. (2020)	https://doi.org/10.14573/altex.2006201
*Saccharomyces cerevisiae* BJ3505 medium and substrate	Morlock and Klingelhöfer (2014)	https://doi.org/10.1021/ac501723j
*Saccharomyces cerevisiae* BJ1991 medium and substrate	Klingelhöfer et al. (2020)	https://doi.org/10.1016/j.aca.2020.05.057
Acetyl-/butyrylcholinesterase solution and substrate	Jamshidi-Aidji and Morlock (2018)	https://doi.org/10.1021/acs.analchem.8b03407
Heraeus Pico 17 Centrifuge	Thermo Scientific, Waltham, MA, USA	www.thermofisher.com
HPTLC system consisting of Automated TLC Sampler 4, Automated Developing Chamber 2, TLC Visualizer, Derivatizer, TLC Immersion Device and BioLuminizer	CAMAG, Muttenz, Schweiz	www.camag.com
Alternative open source OCLab3 system	open source	https://github.com/OfficeChromatography/OCLab3
KIS polypropylene box, 26.5 cm × 16 cm × 10 cm	ABM, Wolframs–Eschenbach, Germany	https://bauhaus.ch/de/kis-bi-box-xs-schwarz-66745701
Laboratory oven	Memmert, Schwabach, Germany	www.memmert.com
Standalone HPLC pump 515	Waters, Eschborn, Germany	www.waters.com
Dionex Ultimate HPLC system equipped with binary pump (HPG-3200SD), autosampler (WPS-3000TXRS), column oven (TCC-3000RS) and diode array detector (DAD-3000RS)	Dionex Softron, Germering, Germany	www.thermofisher.com
Q Exactive Plus Hybrid Quadrupole-Orbitrap	Thermo Fisher Scientific, Bellefonte, PA, USA	www.thermofisher.com
50-mL Centrifuge tubes	Isolab, Wertheim, Germany	https://isolab.de/default
2-mL Laboratory tubes, Protein LoBind Tube	Eppendorf, Hamburg, Germany	www.eppendorf.com

### Resource availability

#### Lead contact

Further information and requests for resources and reagents should be directed to and will be fulfilled by the lead contact, Gertrud E. Morlock (gertrud.morlock@uni-giessen.de).

#### Materials availability

This study did not generate new unique reagents.

### Experimental model and subject details

The microorganisms *A.liivibrio fischeri* NRRL-B11177, *Saccharomyces cerevisiae* BJ1991, containing the hAR expression plasmid, *Saccharomyces cerevisiae* BJ3505, containing the hER expression plasmid, PTM™ *Salmonella typhimurium* TA1535/pSK1002 and *Bacillus subtilis* subsp. s*pizizenii* were used for the planar on-surface assays as described.

### Method details

We confirm that the following experiments were conform to relevant regulatory standards.

#### Study sites

Along the coastline of Hurghada and El Gouna, Northern Red Sea, Egypt, the research area of approximately 600 km^2^ (Figure S1) ranges from the reefs of Shaab Umm Usk in the North (27°35.001 N, 33°52.272 E) to the Abu Hashish reefs in the South (27°1.453 N, 33°55.644 E). Large reef complexes are typical for this region, which is marked by islands and a variety of coastal and offshore coral reefs either isolated or grouped (Riegl and Velimirov 1994; Costa 2015). Belonging to one of the most diverse ecosystems worldwide, these Red Sea reef complexes provide a diversity of substrates to rub on (e.g., hard and soft corals, sponges, sand, sea grass beds). Large parts of the Red Sea comprise a shallow coastal shelf. Next to it, the bottom slopes gradually to depths of 500 m average depth is 491 m and the maximum depth is 2850 m (Edwards and Head, 1987). The salinity ranges from 38-39 ppt, but can also rise to 42 ppt (Behairy et al., 1993). For the Gulf of Aqaba an average summer temperature of 21–27°C is given for the upper 200 m (Manasrah et al., 2006). It offers optimal conditions for the emergence of coral reefs and contains a high number of endemic species (Fine et al., 2019). The organism samples were taken by SCUBA diving on the reefs Shaab El Erg and Shaab El Fanous which are valuable meeting points for the dolphins, especially for resting (Kreicker and Ziltener 2017; Fumagalli et al., 2019), but also for socializing (Orbach et al., 2019) and rubbing on selected substrates (Ziltener et al., 2015). Since it is obvious that the reefs with the occurring organisms are regularly visited by the dolphins, it is important to protect these areas. For this reason, the areas have now been declared an Important Marine Mammal Area. However, further work is needed to ensure the full protection of these sites (Marine mammal, 2019).

#### Behavioural data collection

Photo-identification of dolphins by using the mark-recapture method was conducted in a research boat for at least 5 min (Würsig and Würsig 1977). Data were collected including group behaviour, group composition and structure, GPS location, water depth and features of the accessed organism. Secondly, in appropriate situations, a minimum of two SCUBA divers then joined the dolphins underwater and conducted a group follow using video and photo cameras. Individual identification data was completed with whole body photographs that allowed gender and age classifications, the assessment of reproductive state and physical condition (e.g., skin lesions, ectoparasites, *etc*.).

#### Substrate collection

The project was authorized by the Egyptian Ministry of Environment on 22 July 2019 and supervised by the Red Sea National Parks Authorities. The dives off the coast of El Gouna and Hurghada, Egypt, were conducted from 30 July 2019 to 1 August 2019 according to the standards of recreational and scientific diving (VDST, CMAS) and according to Egyptian regulations. Boat surveys were conducted for a minimum of 5 min *ad libitum*. When the conditions were given, two SCUBA divers started to follow a group of dolphins to continuously record underwater behaviour on video. In addition to the dolphin behaviour surveys, independent dives were conducted to collect the three distinct marine organisms visited by the dolphins. During the dives, only those organisms were sampled that were explicitly accessed by the dolphins for rubbing (Table S1 Compilation of the organisms, related to Figures 1 and S1). The initial dive protocol (Brümmer et al., 2009) was adapted to the present sampling, so that it contained the following described steps. First, a photo of the labelled sample tube was taken with an underwater camera (TG-4 with PT-056, Olympus Corporation, Tokyo, Japan) and an underwater torch (DS-21000 NWRUV, DiveSun LED Tauchlampen, Chemnitz, Germany), so it was possible to assign each sample to the corresponding organism. For each sample the depth at which it was taken was noted (Table S1 Compilation of the organisms, related to Figures 1 and S1). For underwater photo documentation, several overview images and several detailed images were taken, both with and without a scale (Figures S3−S5). In this way, each collection position was specified in terms of the exact location visited by the dolphins. Depending on the organism, small pieces of about 3–5 cm each were cut off with scissors or a scalpel (Figure 1 and S3–S5) and transferred to a 50-mL centrifuge tube (Isolab, Wertheim, Germany) filled with sea water. To obtain another specimen for each individual organism, a second sample was imaged taken, and documented in the same way. Additional samples were taken in labeled 2-mL laboratory tubes (Protein LoBind Tube, Eppendorf, Hamburg, Germany). In order to close these small tubes under water, a small air bubble had to be in the tubes. For these samples, the extraction solvent was already added on the boat directly after the dive, without freezing the samples in between. Therefore, the remaining water was removed with a micropipette (Eppendorf) and 1 mL methanol (99.9%; Merck, Darmstadt, Germany) was added (IDs 7–10). The other samples were drained. All sample tubes were put into a cooling box half-filled with ice and salt to freeze them as fast as possible aboard. The box was tightly sealed with a lid. Afterwards the drained samples were prepared in the laboratory at the El Gouna campus of the Technical University of Berlin. Each collected gorgonian coral (G), leather coral (L) and sponge (S) sample was cut with a scalpel into very small pieces and divided with tweezers as homogeneously as possible in 2-mL laboratory tubes. Either methanol (99.9%; IDs 1–6) or *n-*hexane (95%; Tedia Company, Fairfield, OH, USA; IDs 11–16) was added according to the structural consistency of the marine organism as follows: 200, 400 and 800 μL for leather coral, sponge and gorgonian coral, respectively (Table S2 Sample preparation, related to STAR Methods). Note that the volumes were kept as low as possible to avoid concentration/reconstitution steps and to allow small application volumes for analysis. All tubes were stored at −20°C and transported chilled to Germany.

#### Sample preparation

Although the fresh organisms were cut into comparably small pieces for extraction, the surface area of the differently structured substrates and their intrinsic water contents varied, which could influence the extraction yield. Hence, the effect-directed profiling was used for a qualitative comparison only. The gorgonian coral extract was quite clear and dark brown due to the natural dyes contained therein. The leather coral extract was comparatively brighter and more of a suspension. The sponge extract was light-brownish grey and very turbid due to its fine structural consistency. Methanol samples were vortexed and ultrasonicated at almost 100% humidity at 20–25°C for 10 min (Sonorex Digiplus, Bandelin, Berlin, Germany). Each solution was transferred to a 1.5-mL Eppendorf tube (Eppendorf, Hamburg, Germany) and centrifuged for 5 min at 13,000 rpm (Heraeus Pico 17 Centrifuge, Thermo Scientific, Waltham, Massachusetts, USA). The supernatant was transferred into sample vials for HPTLC. Since *n-*hexane was almost evaporated during the transport to Germany, a 200-μL portion *n-*hexane (95%; Chemsolute, Th. Geyer, Renningen, Germany) was added for volume compensation to these samples. As the marine organism contained water, two phases were formed, which were united by the 1:1 dilution with acetone (Merck, for chromatography). In detail, 500, 700 and 750 μL acetone were added to the gorgonian coral, leather coral and sponge extracts, respectively (Table S2 Sample preparation, related to STAR Methods), which were prepared as mentioned.

#### Effect-directed profiling

The extracts were weighed back and the approximate substrate amounts were calculated to be around 1 mg ± 0.4 mg depending on the structure of the organism (Table S2 Sample preparation, related to STAR Methods). The samples (1–10 μL depending on the assay) were automatedly applied (ATS 4, CAMAG, Muttenz, Switzerland). The settings ''fill only programmed volume'' and ''vacuum time 0 s'' were crucial to avoid any volume loss of the sample during the application. The application volume of the boat samples was comparatively higher depending on the substrate (2.4, 3.2 and 3.6 μL instead of 1 μL) to compensate for and adjust to the higher methanol addition (1 mL for all). The development on HPTLC plates silica gel 60 was automatedly performed (ADC 2, CAMAG) with ethyl acetate – methanol – water 15:3:1 (*V*/*V*/*V*). For separation of apolar zones, *n-*hexane – ethyl acetate 3:4 (Figures S10C–S10F) and 1:4 (Figure S10G) were used. Each chromatogram was multi-detected at white light illumination (Vis), 254 and 366 nm (TLC Visualizer 2, CAMAG). For each assay, a respective positive control (PC) was applied on the upper plate edge. The assay suspensions or solutions were piezoelectrically sprayed; if not stated otherwise (Derivatizer, CAMAG). The chromatogram was horizontally placed for incubation in a polypropylene box (27 × 16 × 10 cm, KIS, ABM, Wolframs–Eschenbach, Germany). It was pre-moistened for 30 min, using 35 mL water spread on filter papers aligned on walls and bottom at room temperature. Images at FLD 366 nm or white light illumination were recorded depending on the assay. The bioluminescence images were taken in the BioLuminizer (CAMAG). As an alternative instrumental system, the miniaturized open source OCLab3 system (https://github.com/OfficeChromatography/OCLab3) can be used (Morlock 2021). The following ten assays were applied:

1) For the Gram–negative *Aliivibrio fischeri* bioassay (Klöppel et al., 2008; DIN EN ISO 2009; Klöppel et al., 2013), 150 μL cryostock (NRRL-B11177, strain 7151; Leibniz Institute, DSMZ, Braunschweig, Germany) were cultivated in 20 mL medium in a 100–mL culture flask at 75 rpm and room temperature for 18–24 h according to DIN EN ISO 11348–1. When the green–blue bioluminescence of the bacteria was brilliant, 4 mL bacterial suspension were sprayed on the plate (blue nozzle, level 6). The still humid plate placed in the cabinet of the BioLuminizer (CAMAG). Ten images were recorded over 30 min with an exposure time of 60 s and trigger interval of 3.0 min. Antimicrobials were detected as a dark or bright band on the instantly bioluminescent plate background. Caffeine (0.5, 1.5 and 3 μL/band, 1 mg/mL in methanol) was used as a positive control.
2) For the Gram–positive *Bacillus subtilis* bioassay (Jamshidi-Aidji and Morlock 2016; Chandana and Morlock 2021), 100 μL cryostock (BGA, DSM 618 strain, spores, Merck, Darmstadt, Germany) were cultivated in 20 mL 2.3% Müller–Hinton broth with an optical density of ca. 0.8 at 600 nm. Thereof, 3.5 mL bacteria suspension were sprayed (red nozzle). Incubation at 37°C followed for 2 h in an oven (Memmert, Schwabach, Germany). Then, 500 μL 0.2% DPBS–buffered MTT solution was sprayed and incubated at 37°C for 45 min, followed by drying (50°C, 5 min, Plate Heater, CAMAG). Colourless (white) antibacterial bands were obtained on a purple background. Tetracycline (0.5, 1.5 and 3 μL/band, 0.005 mg/mL in ethanol) was used as positive control.
3/4) For the acetyl-/butyrylcholinesterase inhibition assays (Jamshidi-Aidji and Morlock 2018; Morlock et al., 2021), 1 mL Tris–HCl buffer (pH 7.8, 0.05 M, for prewetting) were sprayed (green nozzle), followed by 3 mL enzyme solution (6.6 U/mL acetylcholinesterase from *Electrophorus electricus,* or 3.3 U/mL butyrylcholinesterase from equine serum, both in Tris-HCl buffer, Sigma–Aldrich–Fluka, Steinheim, Germany). Incubation at 37°C followed for 25 min. Piezoelectric spraying of 0.75 mL of a 1:2 mixture of ethanolic α–naphthyl acetate solution and aqueous Fast Blue B salt solution (each 3 mg/mL, red nozzle) led to colourless (white) inhibition bands on a purple background. Rivastigmine (2, 4 and 8 μL/band, 0.1 mg/mL in methanol) was used as a positive control.
5/6) For the planar yeast estrogen screen (pYES) bioassay (Morlock and Klingelhöfer 2014), 1 mL cryostock (1 × 10^8^ *Saccharomyces cerevisiae* BJ3505 cells genetically modified by McDonnell et al., 1991) was added to 29 mL culture medium in a 100-mL glass culture flask, followed by incubation at 30°C by shaking at 100 rpm for 20–22 h. The cell culture adjusted to 0.8 × 10^8^ mL^−1^ cells (2.8 mL) was piezoelectrically sprayed (Derivatizer, CAMAG, red nozzle, spraying level 6) and incubated at 30°C and 100% humidity for 3 h. After drying in a cold stream of air for 4 min, the substrate solution (2 mg 4-methylumbelliferyl-β-D-galactopyranoside, 100 μL dimethyl sulfoxide, 3 mL citrate buffer pH 12) was sprayed on. After the second incubation (1 h, 37°C), the detection at FLD 366 nm (Reprostar 3, CAMAG) showed estroge*n-*like compounds as blue fluorescent bands. The estrogen 17β-estradiol (E2, 1 μL, 100 ng/mL in ethanol) was used as a positive control. For the planar yeast antagonistic estrogen screen (pYAES) bioassay, the estrogen 17β-estradiol (2 ng/mL; 5 μL applied) was applied, partially overlapping (e.g., at 7–8 mm of the 12–mm band) along each track as 1 × 70 mm area (FreeMode option, winCATS software), before the assay application.
7/8) For the planar yeast androgen screen (pYAS) bioassays (Klingelhöfer et al., 2020), the workflow was analogously performed to the pYES bioassay, except for the use of *Saccharomyces cerevisiae* BJ1991 cells containing the human AR expression plasmid (Xenometrix, Allschwil, Switzerland) and an incubation of only 4 h. Testosterone (1 μL, 0.75 μg/mL in methanol) was used as a positive control. For the planar yeast antagonistic androgen screen (pYAAS) bioassay, the androgen testosterone (4 μL, 1.5 μg/mL in methanol) was applied, partially overlapping (e.g., at 7–8 mm of the 12–mm band) along each track as a 1 × 70 mm area (FreeMode option, winCATS software) before the assay application.
9) For the genotoxic bioassay (Meyer et al., 2020), 20 μL cryostock (PTM™ *Salmonella typhimurium* TA1535/pSK1002, Trinova Biochem, Giessen, Germany) were cultivated in 35 mL lysogeny broth for 16 h to reach an optical density of ca. 0.2 at 660 nm after re-suspension. The chromatogram was immersed (immersion speed 3.5 cm/s, for 3 s) in the *Salmonella* suspension and incubated at 37°C for 3 h. Alternatively, spraying was used. After plate drying, the workflow was the same as for the pYES bioassay. 4-NQO (1 ng/μL, 1 μL in methanol) was manually applied as a positive control.
10) For the 2,2-diphenyl-1-picrylhydrazyl (DPPH⋅) assay (Krüger et al., 2018), 4 mL 0.04% methanolic DPPH⋅ solution was sprayed (green nozzle, level 4) to instantly generate yellow bands on a purple background. The plate image was inspected again on the next day due to signal intensity increase. Gallic acid (0.5, 1.3 and 2 μL/band, 0.1 mg/mL in methanol) was used as a positive control.

#### Characterization by HPTLC–HESI-HRMS

Higher sample volumes were applied twofold (as listed in respective figure legend). Plates were prewashed (Morlock 2014). After development, each HPTLC plate was cut in two identical halves, one was subjected to the respective assay and the other was used for the HRMS recording. For the latter, the positions were marked on the chromatogram with reference to the active zones on the other plate half. The marked zones were eluted with methanol for 1 min at a flow rate of 0.2 mL/min provided by a Dionex Ultimate LPG-3400XRS quaternary pump (Dionex Softron) using an elution head–based interface (TLC–MS Interface 2, CAMAG). The latter was coupled to a Q Exactive Plus Hybrid Quadrupole Orbitrap mass spectrometer (Thermo Fisher Scientific) equipped with an Ion Max HESI II source operated with the following settings: spray voltage ±3.5 kV, capillary temperature 270°C, sheath gas 20 arbitrary units, aux gas 10 arbitrary units, S–Lens RF level 50 and probe heater temperature 200°C. Full scan mass spectra (*m*/*z* 100–1000) were recorded with a resolution of 280,000 (FWHM at *m/z* 200), AGC target 1e6, maximum inject time 200 ms and the lock masses 413.26623 (diisooctyl phthalate) and 301.14103 (dibutyl phthalate) in the positive ionization mode, and 255.23295 (palmitic acid) and 283.26425 (stearic acid) in the negative ionization mode. From each analyte spectrum, a plate background spectrum was subtracted using Xcalibur 3.0.63 with Foundation 3.0 SP2 software (Thermo Fisher Scientific).

#### Characterization by NP-HPTLC−bioassay−RP-HPLC−PDA−HESI-MS

According to the latest workflow (Schreiner and Morlock 2021) bioactive zones were eluted (TLC-MS Interface, CAMAG) with 10% aqueous methanol (MS-grade, Honeywell, Morristown, NJ, USA) provided by a standalone pump (515 HPLC pump, Waters, Eschborn, Germany) at a flow rate of 0.1 mL/min. The eluted zone was transferred through a biocompatible inline-filter (IDEX Health & Science, Oak Harbor, WA, USA) containing a PEEK frit (0.5 μm, Techlab, Braunschweig, Germany) to a two-position switching-valve (MXT-Series PD715-000, Rheodyne IDEX Health & Science), where the analytes were trapped on a defender guard/pre-column (Accucore RP-MS, 10 mm × 2.1 mm, 2.6 μm, Thermo Scientific, Bellefonte, PA, USA). By switching, trapped analytes were guided to an Acquity UPLC System (Waters, Eschborn, Germany) equipped with a quaternary solvent manager, sample manager, column oven (thermostated at 40°C), photodiode array detector and a single quadrupole mass spectrometer. Binary gradient (A: methanol, B: 2.5 mM ammonium acetate, pH 4.5 adjusted with acetic acid) flushed the precolumn and transferred the analytes to the main RP column (Accucore RP-MS, 100 mm × 2.1 mm, 2.6 μm, Thermo Scientific) for orthogonal separation. The photodiode array detector (190–400 nm) was operated with a deuterium lamp, and the MS instrument according to the following conditions: polarity switching mode with cone voltage ±10 V, sampling frequency 5 Hz, ESI probe temperature 600°C, source temperature 120°C and scan range *m/z* 50–1000. Instruments were controlled by MassLynx V4.2 software (Waters). Mass spectra were depicted after a plate blank subtraction, recorded at a position comparable to the analyte.

### Quantification and statistical analysis

All experiments were repeatedly performed and confirmed the imaging results or mass signals. Quantification and statistical analysis are not applicable.

### Additional resources

Additional resources were not applicable.

## Data Availability

This study did not generate new codes. Any additional information required to reanalyze the data reported in this paper is available from the lead contact upon request.

## Supporting citations

The following reference appears in the supplemental information: Ahmed et al. (2019); Blunt et al. (2008); Bowden et al. (1979); Chang et al. (2020); Chung et al. (2010), Chung et al. (2013); Chung et al. (2014a); Chung et al. (2014b); Chung et al. (2014c); Edwards and Head (1987); Kobayashi et al. (1979); Sung et al. (2007a); Sung et al. (2009); Tatli et al. (2008); Yin et al. (2020); Zubair et al. (2016); Chung et al. (2011); Loya et al. (1997)

## Footnotes

Supplemental information can be found online at https://doi.org/10.1016/j.isci.2022.104271.
