# Supplementary material for: Evidence that Indo-Pacific bottlenose dolphins self-medicate with invertebrates in coral reefs
Source: iScience. 2022 May 19;25(6):104271. doi: 10.1016/j.isci.2022.104271 (PMC9236899; doi:10.1016/j.isci.2022.104271)
Supplement: Document S1. Figures S1–S13 and Tables S1–S6 [file mmc1.pdf]

iScience, Volume *25*

Supplemental information

Evidence that Indo-Pacific bottlenose dolphins self-medicate with invertebrates in coral reefs

Gertrud E. Morlock, Angela Ziltener, Sascha Geyer, Jennifer Tersteegen, Annabel Mehl, Tamara Schreiner, Tamer Kamel, and Franz Brümmer

Supplementary Information

Table S1 Compilation of the organisms explicitly accessed by the dolphins for repeated rubbing behaviour. Sampling data on the three different organisms collected from 30th July 2019 to 1st August 2019 according to the standards of recreational and scientific diving (VDST, CMAS) and according to Egyptian regulations, Related to Figure 1.

Marine organism	ID	Sample place	Depth [m]
Rumphella aggregata	G1	Shaab El Fanous West	9.5
	G2	Shaab El Fanous East	12.0
	G3	Shaab El Erg	10.5
	G4	Shaab El Erg	10.6
	G5	Shaab El Erg	10.8
	G6	Shaab El Erg	10.0
	G7	Shaab El Erg	10.5
	G8	Shaab El Erg	10.6
	G9	Shaab El Erg	10.8
	G10	Shaab El Erg	10.0
Ircinia sp.	S1	Shaab El Fanous East	12.7
	S2	Shaab El Fanous East	12.8
	S3	Shaab El Erg	8.3
	S4	Shaab El Erg	9.7
	S5	Shaab El Erg	7.8
	S6	Shaab El Erg	7.1
	S7	Shaab El Erg	8.3
	S8	Shaab El Erg	9.7
	S9	Shaab El Erg	7.8
	S10	Shaab El Erg	7.1
Sarcophyton sp.	L1	Shaab El Erg	10.5
	L2	Shaab El Erg	12.0
	L3	Shaab El Erg	9.2
	L4	Shaab El Erg	9.4
	L5	Shaab El Erg	9.8
	L6	Shaab El Erg	9.2
	L7	Shaab El Erg	9.2
	L8	Shaab El Erg	9.4
	L9	Shaab El Erg	9.8
	L10	Shaab El Erg	9.2

Table S2 Sample preparation of the three different organisms collected. Related to Figure 1.

Sponge ID	Treated in	Same as	Tube no.	Extraction solvent	Solvent volume [µL]	ad volume[1] [µL]	Sample weight [mg][2]
S1			1	Methanol	400	-	1352
	Lab		2	Methanol	400	-	1242
S11			3	*n*-Hexane	400	1350	1465
S2			1	Methanol	400	-	1738
	Lab		2	Methanol	400	-	1389
S12			3	*n*-Hexane	400	1350	1122
S3			1	Methanol	400	-	1216
	Lab	S7	2	Methanol	400	-	1463
S13			3	*n*-Hexane	400	1350	1240
S4			1	Methanol	400	-	875
	Lab	S8	2	Methanol	400	-	1088
S14			3	*n*-Hexane	400	1350	949
S5			1	Methanol	400	-	1139
	Lab	S9	2	Methanol	400	-	1314
S15			3	*n*-Hexane	400	1350	1131
S6			1	Methanol	400	-	1369
	Lab	S10	2	Methanol	400	-	1452
S16			3	*n*-Hexane	400	1350	1463
S7	Boat			Methanol	1000	-	600
S8	Boat			Methanol	1000	-	897
S9	Boat			Methanol	1000	-	970
S10	Boat			Methanol	1000	-	897
**Gorgonian coral ID**	**Treated in**	**Same as**	**Tube no.**	**Extraction solvent**	**Solvent volume [µl]**	**After addition[3] [µL]**	**Sample weight [mg][2]**
G1			1	Methanol	800	-	1224
	Lab		2	Methanol	800	-	1258
G11			3	*n*-Hexane	800	1500	892
G2			1	Methanol	800	-	1285
	Lab		2	Methanol	800	-	1090
G12			3	*n*-Hexane	800	1500	671
G3			1	Methanol	800	-	1088
	Lab	G7	2	Methanol	800	-	1138
G13			3	*n*-Hexane	800	1500	730
G4			1	Methanol	800	-	1231
	Lab	G8	2	Methanol	800	-	1379
G14			3	*n*-Hexane	800	1500	685
G5			1	Methanol	800	-	1146
	Lab	G9	2	Methanol	800	-	1016
G15			3	*n*-Hexane	800	1500	605
G6			1	Methanol	800	-	1043
	Lab	G10	2	Methanol	800	-	1049
G16			3	*n*-Hexane	800	1500	458
G7	Boat			Methanol	1000	-	813
G8	Boat			Methanol	1000	-	967
G9	Boat			Methanol	1000	-	735
G10	Boat			Methanol	1000	-	1046

Leather coral ID	Treated in	Same as	Tube no.	Extraction solvent	Solvent volume [µl]	After addition[4] [µL]	Sample weight [mg][2]
L1			1	Methanol	200	-	853
	Lab		2	Methanol	200	-	793
L11			3	*n*-Hexane	200	1100	936
L2			1	Methanol	200	-	899
	Lab		2	Methanol	200	-	952
L12			3	*n*-Hexane	200	1100	915
L3			1	Methanol	200	-	925
	Lab	L7	2	Methanol	200	-	887
L13			3	*n*-Hexane	200	1100	823
L4			1	Methanol	200	-	786
	Lab	L8	2	Methanol	200	-	984
L14			3	*n*-Hexane	200	1100	915
L5			1	Methanol	200	-	1002
	Lab	L9	2	Methanol	200	-	933
L15			3	*n*-Hexane	200	1100	1180
L6			1	Methanol	200	-	770
	Lab	L10	2	Methanol	200	-	933
L16			3	*n*-Hexane	200	1100	722
L7	Boat			Methanol	1000	-	972
L8	Boat			Methanol	1000	-	994
L9	Boat			Methanol	1000	-	687
L10	Boat			Methanol	1000	-	843

[1] 200 µL *n*-Hexane and 750 µL acetone were added.

[2] The extractant volumes in the Eppendorf tubes were 200, 400, 800 and 1000 µL of *n*-hexane or methanol as specified. In Germany, the weight of the samples was determined approximately: three empty Eppendorf tubes with the respective extractant were weighed and averaged. This mean value was subtracted from the respective sample tube weight.

[3] 200 µL *n*-Hexane and 500 µL acetone were added.

[4] 200 µL *n*-Hexane and 700 µL acetone were added.

Table S3 Ten different assays applied for effect-directed profiling. Organisms used in the effect-directed assays and respectively generated responses for detection of bioactive metabolites in the three selected substrates. Related to Figure 2.

**Biological assay**		**Final response of the band**	**Interpretation**
Gram-negative bacteria	*Aliivibrio fischeri*	Dark	Antimicrobials and bioactive compounds<br>- Reduction of bioluminescence related to decrease of energetic cell metabolism
		Bright	- Increase of bioluminescence related to improved energetic cell metabolism
	Salmonella typhimurium TA1535/pSK1002	Blue fluorescent	Genotoxic effect
Gram-positive bacteria	*Bacillus subtilis*	Colourless	Antimicrobials
Yeast cells	*Saccharomyces cerevisiae* BJ3505	Blue fluorescent	Estrogen-like effect
		Reduction of blue fluorescence	Antiestrogen-like effect
	Saccharomyces cerevisiae BJ1991	Blue fluorescent	Androgen-like effect
		Reduction of blue fluorescence	Antiandrogen-like effect
**Biochemical assay**			
Enzymes	Acetylcholinesterase inhibition assay	Colourless	Neurotoxins or inhibitors of the degrading of the neurotransmitter acetylcholine in the nerve synapses, thus improving cholinergic neurotransmission
	Butyrylcholinesterase inhibition assay	Colourless	Neurotoxins or inhibitors decreasing the increased number of neuritic plaques in demented brains, acting no*n*-specific in plasma and tissue
**Chemical assay**			
Chemical	2,2-Diphenyl-1-picrylhydrazyl (DPPH•)	Yellow	Radical scavengers

Table S4 Compilation of activities described in literature. Bioactive metabolites already isolated and described for the three different marine organisms. Related to Figures 3−5.

Species	Substance	Molecular formula	Weight (Da)	Effect	Literature
***Ircinia* sp.**	7-Methyl-9-oxo-dec-7-eneoic acid	$C_{11}H_{18}O_3$	198.26	Active in Alzheimer's diseases	(Tatli et al. 2008)
	2-Hexaprenylhydroquinone	$C_{36}H_{54}O_2$	518.81	Reverse transcriptase and HIV-integrase inhibitor, bacteriostatic	(Bifulco et al. 1995; Loya et al. 1997)
	Pentaprenylhydroquinone 4-sulfate	$C_{31}H_{46}O_5S$	530.77	Neuropeptide Y receptor, tyrosine protein kinase and HIV-integrase inhibitor	(Bifulco et al. 1995)
	Hexaprenylhydroquinone 4-sulfate	$C_{36}H_{54}O_5S$	598.89	Neuropeptide Y receptor, tyrosine protein kinase and HIV-integrase inhibitor	
	Heptaprenylhydroquinone 4-sulfate	$C_{41}H_{62}O_5S$	667.01	Neuropeptide Y receptor, tyrosine protein kinase and HIV-integrase inhibitor	
	Variabilin	$C_{25}H_{34}O_4$	398.54	Antibacterial	(Faulkner 1973)
	Fasciculatin	$C_{25}H_{34}O_4$	398.54	Inosine monophosphate dehydrogenase inhibitor Moderately cytotoxic	(Cafieri et al. 1972; Rifai et al. 2005)
	Palinurin	$C_{25}H_{34}O_4$	398.54	Cytotoxic	(Martí et al. 2003)
	Tedanolide C	$C_{32}H_{50}O_{11}$	610.73	Cytotoxic	(Blunt et al. 2008)
	Irciniasulfonic acid B1	$C_{36}H_{65}NO_6S$	639.97	Reversing multi-drug resistance in cancer cells	
	Irciniasulfonic acid B2	$C_{34}H_{63}NO_6S$	613.93	Reversing multi-drug resistance in cancer cells	
	Irciformonin C	$C_{23}H_{34}O_7$	422.51	Moderately cytotoxic	
	Irciformonin D	$C_{23}H_{34}O_7$	422.51	Moderately cytotoxic	
	Ircinolin A	$C_{21}H_{34}O_6$	382.49	Cytotoxic	(Mioso et al. 2017)
	15-Acetylirciformonin B	$C_{24}H_{34}O_6$	418.52	Cytotoxic	
	10-Acetylirciformonin B	$C_{24}H_{34}O_6$	418.52	Cytotoxic	
	Irciformonin B	$C_{22}H_{32}O_5$	376.49	Cytotoxic	
	Irciformonin F	$C_{22}H_{32}O_5$	376.49	Cytotoxic	
***Rumphella* sp.**	Fucoxanthin	$C_{42}H_{58}O_6$	658.91	Cytotoxic	(Alarif 2012)
	5α,8α-Epidioxyergosta-6,9(11)-diene-3β-ol	$C_{28}H_{44}O_3$	428.33	Moderately cytotoxic	(Yin et al. 2020)
	Antipacid A	$C_{15}H_{24}O_3$	252.17	Anti-inflammatory	(Chang et al. 2020)
	Rumphellolide L	$C_{30}H_{48}O_4$	472.36	Anti-inflammatory	
	Rumphellaoic acid A	$C_{15}H_{24}O_2$	236.35	Anti-inflammatory	(Chung et al. 2014a)
	Rumphellol A	$C_{15}H_{24}O_2$	236.35	Anti-inflammatory	(Chung et al. 2014c)
	Rumphellol B	$C_{17}H_{30}O_2$	266.22	Anti-inflammatory	
	Rumphellaone C	$C_{14}H_{24}O_4$	268.17	Anti-inflammatory	(Chung et al. 2014b)
	2β-Acetoxyclovan-9α-ol	$C_{17}H_{28}O_3$	280.20	Anti-inflammatory	(Chung et al. 2013)
	9α-Acetoxyclovan-2β-ol	$C_{17}H_{28}O_3$	280.20	Anti-inflammatory	
	Rumphellclovane B	$C_{15}H_{24}O_3$	252.17	Anti-inflammatory	(Chung et al. 2011)
	Rumphellaone A	$C_{15}H_{24}O_3$	252.17	Moderately cytotoxic	(Chung et al. 2010)

	Rumphellolide I	$C_{14}H_{22}O_3$	238.32	Anti-inflammatory	(Sung et al. 2009)
	Rumphellatin B	$C_{14}H_{23}ClO_3$	274.78	Antibacterial<br>- *Staphylococcus aureus*	(Sung et al. 2007a)
	Rumphellolide A	$C_{15}H_{24}O_3$	252.17	Antibacterial<br>- *Pseudomonas aeruginosa*	(Sung et al. 2007b)
	Rumphellolide D	$C_{14}H_{22}O_3$	238.32	Antibacterial<br>- *Pseudomonas aeruginosa*<br>- *Vibrio parahaemolyticus*	
	Rumphellolide E	$C_{14}H_{22}O_3$	238.32	Antibacterial<br>- *Escherichia coli,*<br>- *Pseudomonas aeruginosa*	
	Rumphellolide F	$C_{14}H_{22}O_2$	222.23	Antibacterial<br>- *Staphylococcus aureus*<br>- *Vibrio parahaemolyticus*	
***Sarcophyton* sp.**	Sarcrassin A	$C_{22}H_{32}O_5$	376.49	Moderately cytotoxic	(Blunt et al. 2008)
	Sarcrassin B	$C_{22}H_{32}O_6$	392.47	Moderately cytotoxic	
	Sarcrassin C	$C_{23}H_{36}O_6$	408.53	Moderately cytotoxic	
	Sarcrassin D	$C_{23}H_{32}O_6$	404.50	Moderately cytotoxic	
	Sarcrassin E	$C_{21}H_{28}O_5$	360.44	Moderately cytotoxic	
	Bisglaucumlide C	$C_{43}H_{62}O_{10}$	738.95	Moderately cytotoxic	
	Bisglaucumlide D	$C_{43}H_{62}O_{10}$	738.95	Moderately cytotoxic	
	Alloaromadendrene	$C_{15}H_{24}$	204.35	Cytotoxic	(El-Ezz et al. 2017)
	Sarcophine	$C_{20}H_{28}O_3$	316.44	Cytotoxic, antifungal	
	Sarcophytolide	$C_{20}H_{28}O_3$	316.44	Cytotoxic,<br>antimicrobial properties<br>-*Staphylococcus aureus*<br>-*Pseudomonas aeruginosa*<br>-*Candida albicans*<br>-*Saccharomyces cerevisiae*	
	Sarcophytolide B	$C_{20}H_{28}O_3$	316.44	Cytotoxic	
	Sarcophytolide C	$C_{20}H_{28}O_3$	316.44	Cytotoxic	
	Sarcophytolol	$C_{20}H_{34}O_2$	306.48	Cytotoxic	
	Isosarcophytoxide	$C_{20}H_{30}O_2$	302.45	Antimicrobial	(Bowden et al. 1979)
	Sarcophytol B	$C_{20}H_{32}O_2$	304.47	Cytotoxic	(Kobayashi et al. 1979)
	24ε-Methylcholestane-3β,5α,6β,25-tetraol 25-monoacetate	$C_{30}H_{52}O_5$	492.38	Cytotoxic	(Zubair et al. 2016; Ahmed, M.M.A., Albadry, M.A., Ragab, E.A., El-Ghaly, E.M., Kotb, S.E., Khan, S.I., Chittiboyina, A.G. & Khan, I.A. 2019)
	(24S)-Methylcholestane-3β,5α,6β,12β,25-pentol 25-monoacetate	$C_{30}H_{52}O_6$	508.38	Cytotoxic	
	(24S)-Methylcholestane-3β,5α,6β,25-tetraol 25-monoacetate	$C_{30}H_{52}O_5$	492.38	Cytotoxic	
	(24S)-24-Methylcholestane-3β,5α,6β-triol	$C_{28}H_{50}O_3$	434.38	Cytotoxic	
	(24S)-Ergostan-3β,5α,6β,18, 25-pentaol,18,25-diacetate	$C_{31}H_{52}O_7$	536.37	Cytotoxic	
	Sarcoaldesterol A	$C_{30}H_{52}O_4$	476.39	Antibacterial<br>- *Escherichia coli*<br>- *Bacillus megaterium*<br>and antifungal<br>- *Microbotryum violaceum*<br>- *Septoria tritici*	

Sarcoaldesterol B	$C_{28}H_{50}O_4$	450.37	Antibacterial<br>- *Escherichia coli*<br>- *Bacillus megaterium*,<br>and antifungal<br>- *Microbotryum violaceum*<br>- *Septoria tritici*
11α-Acetoxy-16β-methoxy-23,24-dimethylcholest-17(20)-en-3β,5α,6β-triol	$C_{32}H_{54}O_6$	534.39	Cytotoxic
(24R)-Gorgost-25-en-3β,5α,6β,11α-tetraol	$C_{30}H_{50}O_4$	474.37	Cytotoxic
(24S)-11α-Acetoxy-ergost-3β,5α,6β-triol	$C_{30}H_{50}O_5$	490.37	Cytotoxic
(24R)-Methylcholest-7-en-3β,5α,6β-triol	$C_{20}H_{48}O_3$	432.36	Moderately cytotoxic, antiviral<br>- H1N1 IAV
(24S)-24-Methylcholestan-1b,3b,5a,6b,25-pentaol 25-monoacetate	$C_{30}H_{52}O_6$	508.38	Cytotoxic, anti-inflammatory antibacterial<br>- *Staphylococcus aureus*
Gorgostane-1α,3β,5α,6β,11α-pentaol	$C_{30}H_{52}O_5$	492.38	Moderately antibacterial, antifungal
11α-Acetoxycholest-24-en-1α,3β,5α,6β-tetraol	$C_{29}H_{48}O_6$	492.35	Cytotoxic
Sarcopanol A	$C_{30}H_{50}O_6$	506.36	Anti-inflammatory
Sarcomilasterol	$C_{28}H_{48}O_4$	448.68	Anti-osteoporosic
(24S)-24-Methylcholestan-3β,6β,25-triol-25-O-acetate	$C_{30}H_{52}O_4$	476.39	Antibacterial<br>- *Staphylococcus aureus*

Table S5 Assignment of the NP-HPTLC–HESI–HRMS signals of bioactive zones 1–10. Main signals in bold, Related to Figures 4 and S12.

Zone	Sample	Bioactivity	Fig.	Mass signal *m/z*	Assignment	Molecular formula	Δ ppm	Tentative molecule
**1**	G9	Metabolism enhancing	5D	138.0551<br>**160.0370**	$[M1+H]^+$<br>**$[M1+Na]^+$**	$C_7H_7NO_2$	-0.36<br>-0.31	
				182.0789	$[M2+Na]^+$	$C_7H_{13}NO_3$	-0.44	
				220.0614	$[M3+Na]^+$	$C_6H_{15}NO_4S$	0.14	Cyclohexylamine sulfate
				124.0073	$[M4–H]^–$	Unknown	-	
				**181.0718**<br>217.0483	**$[M5–H]^–$**<br>$[M5+Cl]^–$	$C_6H_{14}O_6$	0.00<br>0.51	Hexane-hexol (*e.g.*, sorbitol)
**2**	S10	Metabolism enhancing	5D	**203.0526**<br>215.0327	**$[M6+Na]^+$**<br>$[M6+Cl]^–$	$C_6H_{12}O_6$	-0.25<br>0.29	Glucose
				124.9913	$[M7–H]^–$	$C_2H_6O_4S$	0.56	Ethyl sufate
**4**	S12	Antibacterial	5D	105.0702	$[M8+H]^+$	C_8H_8	-3.52	
				122.0966	$[M9+H]^+$	$C_8H_{11}N$	-1.64	
				173.0421	$[M10+Na]^+$	$C_5H_{10}O_5$	-0.52	
**6**	G16	Metabolism enhancing	5D	243.0621	$[M11–H]^–$	$C_9H_{12}N_2O_6$	0.53	
**8**	S12	Antibacterial	5D	**265.0795**<br>241.0830	**$[M12+Na]^+$**<br>$[M12–H]^–$	$C_{10}H_{14}N_2O_5$	0.08<br>-0.12	
				111.0199	$[M13–H]^–$	$C_4H_4N_2O_2$	0.54	
				287.0885	$[M14–H]^–$	$C_{11}H_{16}N_2O_7$	-0.14	
**9**	S10	Antibacterial, weakly AChE/BChE inhibiting	5D	399.2531<br>421.2349<br>**397.2383**<br>795.4845	$[M15+H]^+$<br>$[M15+Na]^+$<br>**$[M15–H]^–$**<br>$[2M15–H]^–$	$C_{25}H_{34}O_4$	-0.35<br>-0.05<br>-0.03<br>-0.25	Fasciculatin, variabilin, palinurin
**10**	L8	Antibacterial, AChE/BChE inhibiting	5D<br>S12	**339.1931**<br>655.3972<br>315.1966	**$[M16+Na]^+$**<br>$[2M16+Na]^+$<br>$[M16–H]^–$	$C_{20}H_{28}O_3$	0.24<br>-0.40<br>-0.29	Sarcophine, sarcophytolide, sarcophytolide B, sarcophytolide C
				331.1915	$[M16+O–H]^–$	$C_{20}H_{28}O_4$	0.03	
				333.2072	$[M16+H_2O–H]^–$	$C_{20}H_{30}O_4$	-0.11	
				347.1863	$[M16+2O–H]^–$	$C_{20}H_{28}O_5$	0.26	
				**325.2138**<br>641.4181	**$[M17+Na]^+$**<br>$[2M17+Na]^+$	$C_{20}H_{30}O_2$	0.28<br>-0.41	Isosarcophytoxide
				303.2329	$[M18–H]^–$	$C_{20}H_{32}O_2$	0.03	Sarcophytol B
				118.9419	$[M19–H]^–$	Unknown	-	

Table S6 Assignment of the NP-HPTLC– pYES/pYAAS–RP-HPLC–PDA–HESI-MS signals of bioactive zones 11–15. Main signals in bold, Same signals same colour, Related to Figures 5 and S13.

Zone	Sample	Bioactivity	Fig.	Mass signal *m/z*	Assignment	RT [min]	λ_{max} [nm]
**11**	L8	Estrogen-like	6E	315.18	$[M20-H_2O+H]^+$	8.13/8.24	276
				**333.29**	**$[M20+H]^+$**		
				350.27	$[M20+NH_4]^+$		
				355.21	$[M20+Na]^+$		
				371.05	$[M20+K]^+$		
				331.38	$[M20-H]^-$		
				377.32	$[M20+HCOO]^-$		
				391.32	$[M20+H_3C\text{-}COO]^-$		
**12**	L8/L13	Estrogen-like	6F	**229.35**	**$[M21+H]^+$**	7.07	-
				246.07	$[M21+NH_4]^+$		
				267.10	$[M21+K]^+$		
				**375.29**	**$[M22+H]^+$**	8.24	226
				392.46	$[M22+NH_4]^+$		
				397.08	$[M22+Na]^+$		
				413.42	$[M22+K]^+$		
				299.28	$[M23-H_2O+H]^+$	8.54	231
				**317.20**	**$[M23+H]^+$**		
				334.43	$[M23+NH_4]^+$		
				339.12	$[M23+Na]^+$		
				355.15	$[M23+K]^+$		
				315.24	$[M23-H]^-$		
				333.29	$[M24-H_2O+H]^+$	8.75	-
				373.07	$[M24+Na]^+$		
				389.23	$[M24+K]^+$		
**13**	L8/G11	Estrogen-like	6G	331.27	$[M25-2H_2O+H]^+$	6.72/7.07	-
				349.32	$[M25-H_2O+H]^+$		
				**366.36**	**$[M25]^+$**		
				384.29	$[M25+NH_4]^+$		
				389.16	$[M25+Na]^+$		
				405.19	$[M25+K]^+$		
				365.35	$[M25-H]^-$		
				401.20	$[M25+Cl]^-$		
				411.27	$[M25+HCOO]^-$		
				425.27	$[M25+H_3C\text{-}COO]^-$		
				313.15	$[M26-2H_2O+H]^+$	7.44	-
				331.27	$[M26-H_2O+H]^+$		
				**349.32**	**$[M26+H]^+$**		
				366.36	$[M26+NH_4]^+$		
				387.20	$[M26+K]^+$		
				347.23	$[M26-H]^-$		
				393.28	$[M26+HCOO]^-$		
				**380.61**	**$[M27+H]^+$**		-
				403.42	$[M27+Na]^+$		
				419.12	$[M27+K]^+$		
				415.20	$[M27+Cl]^-$		
				425.08	$[M27+HCOO]^-$		
				439.01	$[M27+H_3C\text{-}COO]^-$		

				297.00<br>315.05<br>**332.15**<br>350.27<br>355.27<br>370.98	$[M20-2H_2O+H]^+$<br>$[M20-2H_2O+H]^+$<br>**$[M20]^+$**<br>$[M20+NH_4]^+$<br>$[M20+Na]^+$<br>$[M20+K]^+$	8.12/8.23	275
				299.28<br>**317.20**<br>334.24<br>338.99<br>355.15<br>655.45	$[M23-H_2O+H]^+$<br>**$[M23+H]^+$**<br>$[M23+NH_4]^+$<br>$[M23+Na]^+$<br>$[M23+K]^+$<br>$[2M23+Na]^+$	8.54	225
**14**	L8	Antiandrogenic	7E	331.33<br>**349.38**<br>366.36<br>387.26<br>347.36	$[M27-H_2O+H]^+$<br>**$[M27+H]^+$**<br>$[M27+NH_4]^+$<br>$[M27+K]^+$<br>$[M27-H]^-$	7.44	-
				403.16<br>419.44<br>415.32<br>425.02<br>439.33	$[M28+Na]^+$<br>$[M28+K]^+$<br>$[M28+Cl]^-$<br>$[M28+HCOO]^-$<br>$[M28+H_3C\text{-}COO]^-$		-
				315.18<br>**332.34**<br>350.08<br>355.08<br>371.11	$[M21-2H_2O+H]^+$<br>**$[M21]^+$**<br>$[M21+NH_4]^+$<br>$[M21+Na]^+$<br>$[M21+K]^+$	8.12/8.23	275
				299.28<br>**317.14**<br>334.31<br>339.18<br>355.34<br>655.45	$[M23-H_2O+H]^+$<br>**$[M23+H]^+$**<br>$[M23+NH_4]^+$<br>$[M23+Na]^+$<br>$[M23+K]^+$<br>$[2M23+Na]^+$	8.53	225
**15**	L13	Antiandrogenic	7F	331.20<br>349.26<br>**366.23**<br>384.29<br>389.35<br>405.32<br>365.22<br>401.33<br>411.27<br>425.14	$[M25-2H_2O+H]^+$<br>$[M25-H_2O+H]^+$<br>**$[M25]^+$**<br>$[M25+NH_4]^+$<br>$[M25+Na]^+$<br>$[M25+K]^+$<br>$[M25-H]^-$<br>$[M25+Cl]^-$<br>$[M25+HCOO]^-$<br>$[M25+H_3C\text{-}COO]^-$	6.73/7.09	-
				299.28<br>**317.14**<br>334.31<br>339.25<br>355.34<br>655.51	$[M23-H_2O+H]^+$<br>**$[M23+H]^+$**<br>$[M23+NH_4]^+$<br>$[M23+Na]^+$<br>$[M23+K]^+$<br>$[2M23+Na]^+$	8.51	208

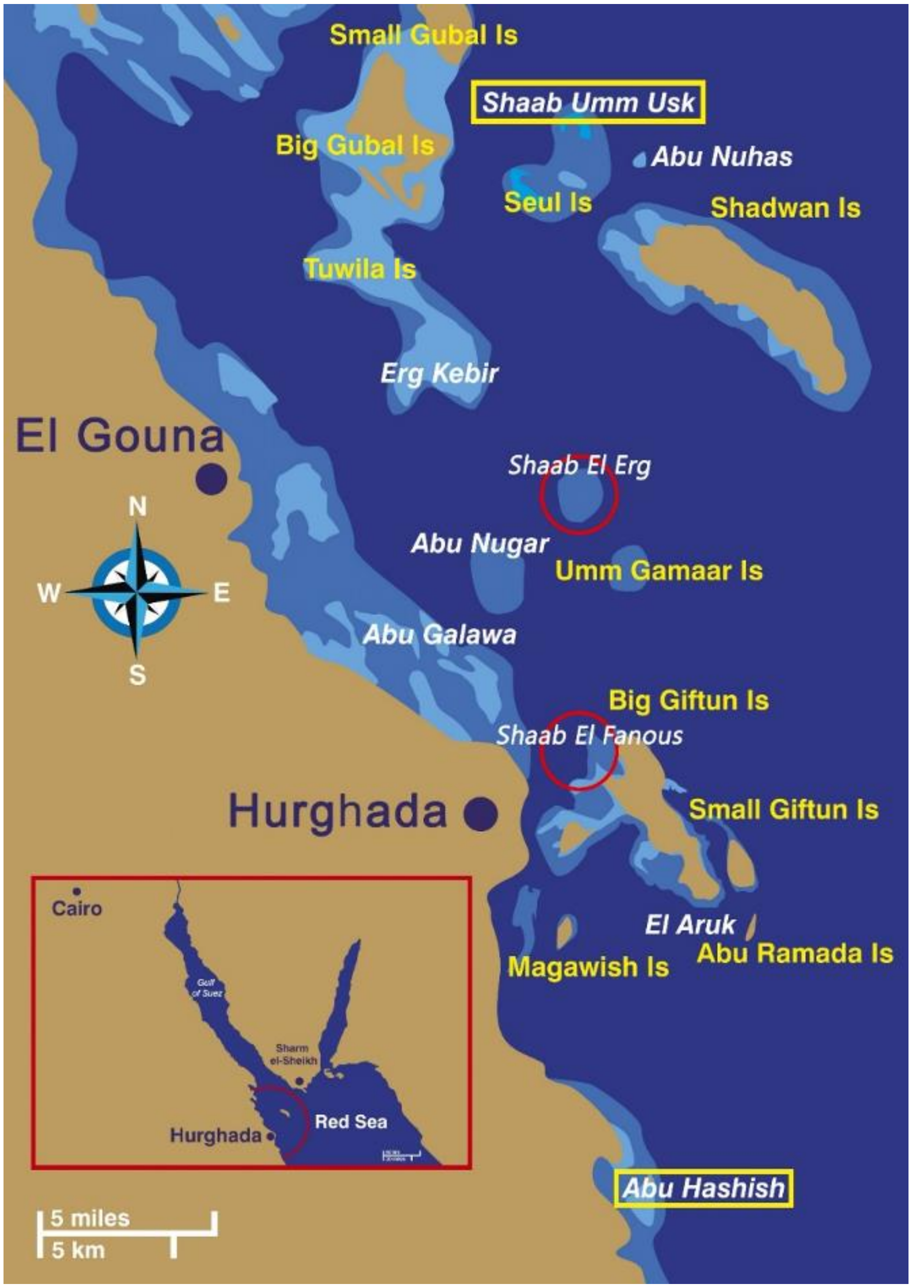

Figure S1. Study site of sampling

Study map of the Indo-Pacific bottlenose dolphins around Hurghada in the Northern Red Sea, Egypt ranges from the reefs of Shaab Umm Usk in the North to the Abu Hashish reefs in the south (yellow boxes). Organism samples were taken at the two reef sites Shaab El Erg and Shaab El Fanous (red circles), which are regularly visited by the dolphins for resting, socializing and rubbing, Related to Figure 1.

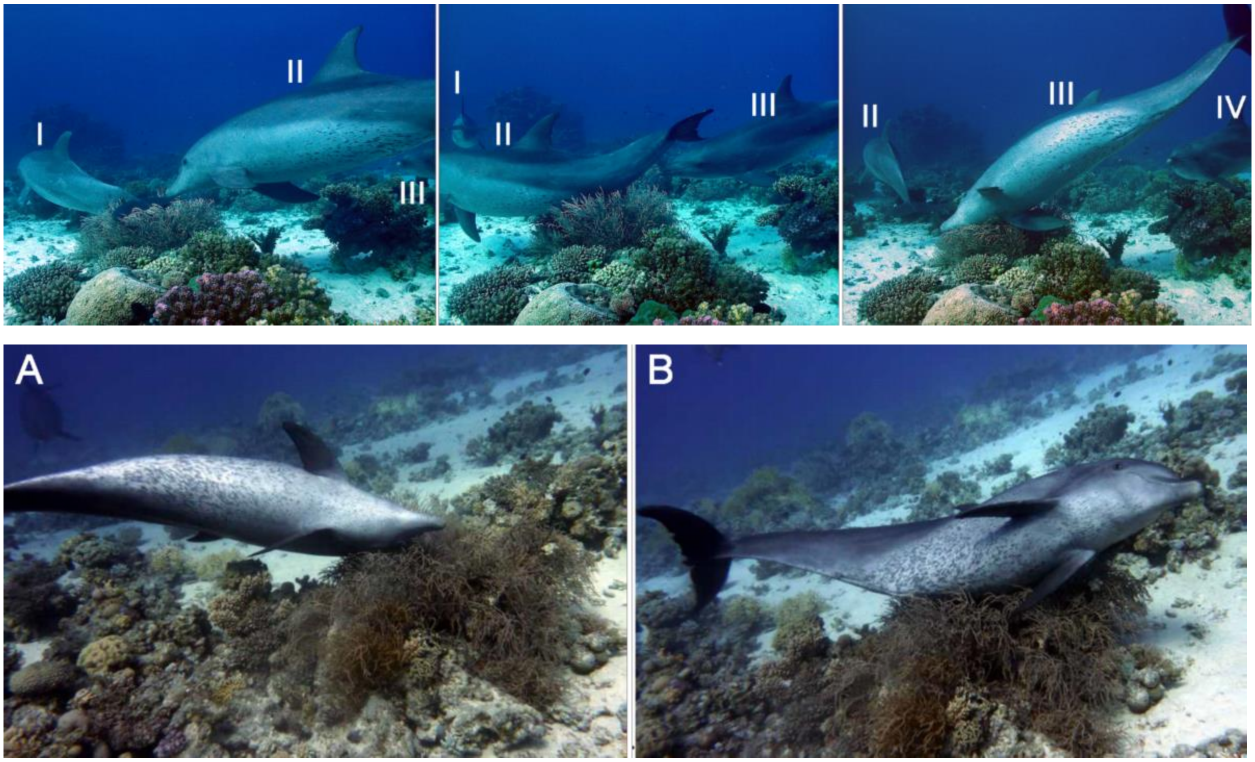

Figure S2. Rubbing behaviour of a group of Indo-Pacific bottlenose dolphins (I–IV), queuing up to rub against the gorgonian coral (gorgoning)

Dolphins queue up behind each other to wait their turn: (A) An Indo-Pacific bottlenose dolphin glides towards and (B) rubs its skin on the gorgonian coral *Rumphella aggregata* as documented in Shaab El Erg and Shaab El Fanous, Red Sea, Egypt (details in Videos S1 and S2). The individual either rubs its ventral, lateral or dorsal body part on the gorgonian coral; its head, pectoral fins and fluke often touch the gorgonian too. This is often repeated so that all body areas are rubbed. When in groups, dolphins queue up behind each other to wait their turn for their next approach. In general, the soft gorgonian coral polyps start to close and retract when dolphins rub on them. This inherent coral protection mechanism can support abrasion, as it allows the dolphins' skin to come into contact with secondary metabolites. A larger mucus secretion from the branches of the coral during the rubbing behaviour has been observed, Related to Figure 1.

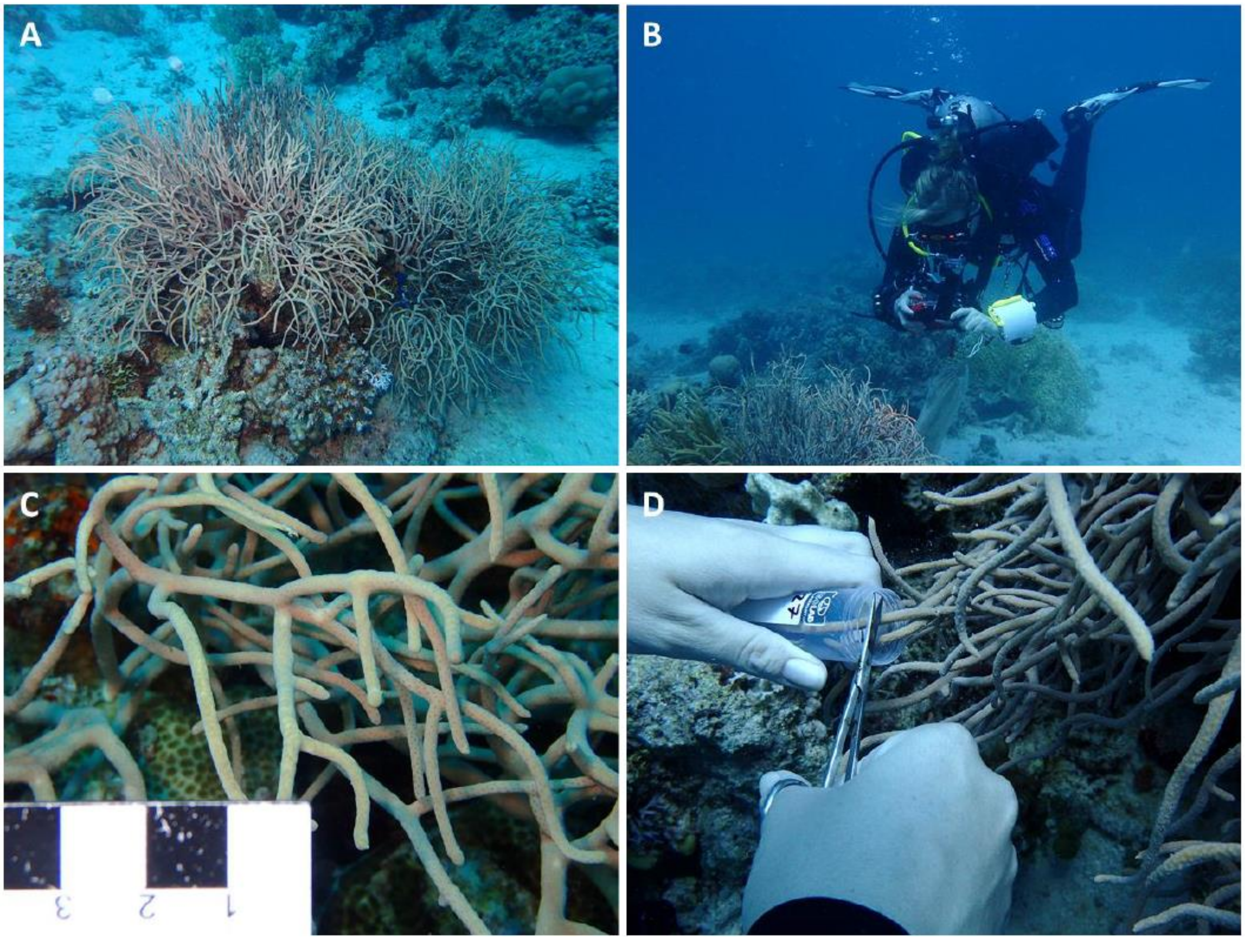

Figure S3. Underwater photo documentation of the gorgonian coral sampling

(A) Individual gorgonian coral *Rumphella aggregata*, (B) overview image, (C) detail image with scale bar and (D) sampling process, Related to Figure 1. Scientific diver Jennifer Tersteegen consents to the use of the image.

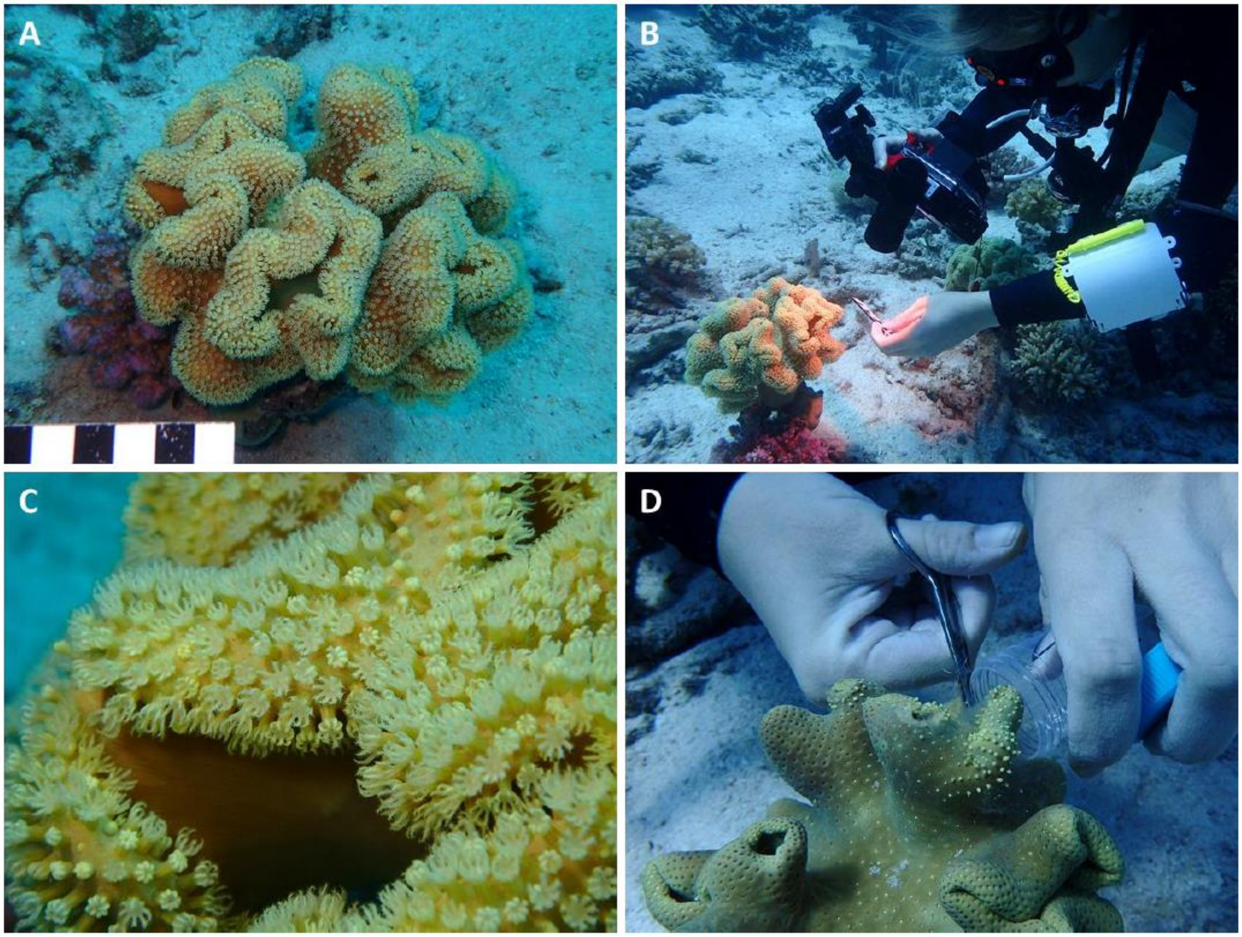

Figure S4. Underwater photo documentation of the leather coral sampling

(A) Individual leather coral *Sarcophyton* sp. with scale bar, (B) overview image, (C) detail image and (D) sampling process, Related to Figure 1. Scientific diver Jennifer Tersteegen consents to the use of the image.

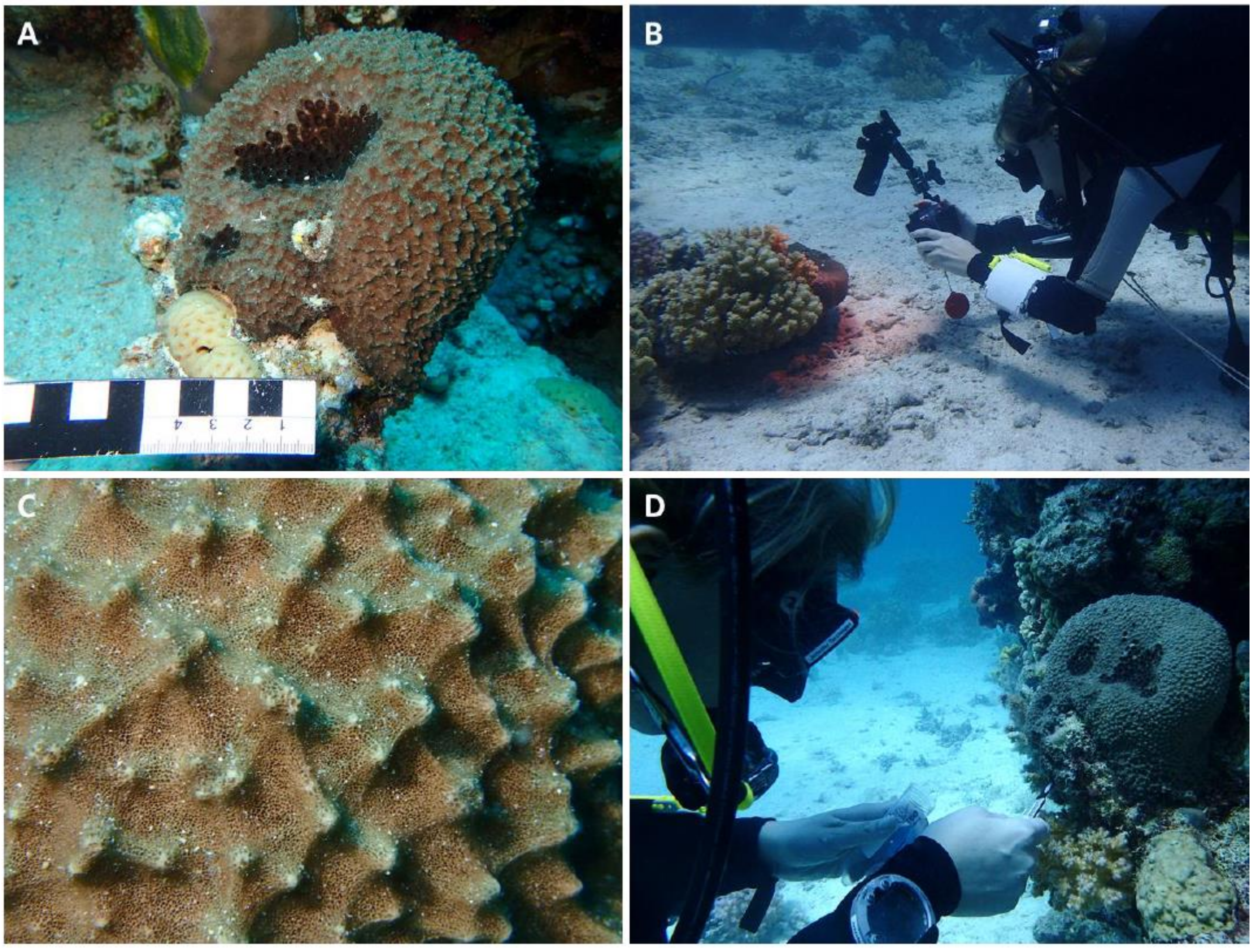

Figure S5. Underwater photo documentation of the sponge sampling

(A) Individual sponge *Ircinia* sp. with scale bar, (B) overview image, (C) detail image and (D) sampling process, Related to Figure 1. Scientific diver Jennifer Tersteegen consents to the use of the image.

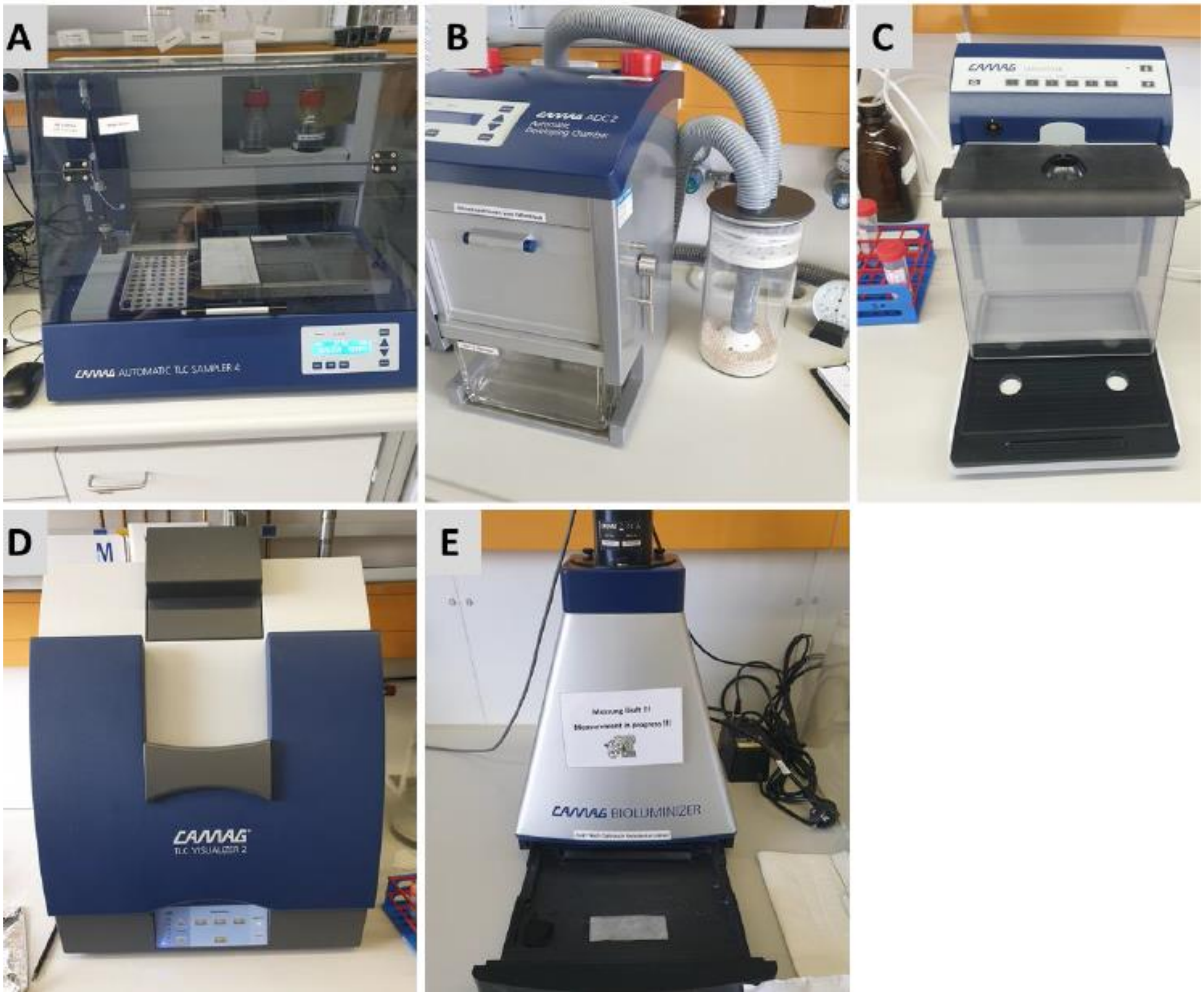

Figure S6. HPTLC instrumentation used for the effect-directed profiling

(A) Application, (B) development, (C) piezoelectric spraying of assay solutions or suspensions, (D) UV/Vis/FLD detection and (E) bioluminescence detection; all in operation in Video S6, Related to Figure 2.

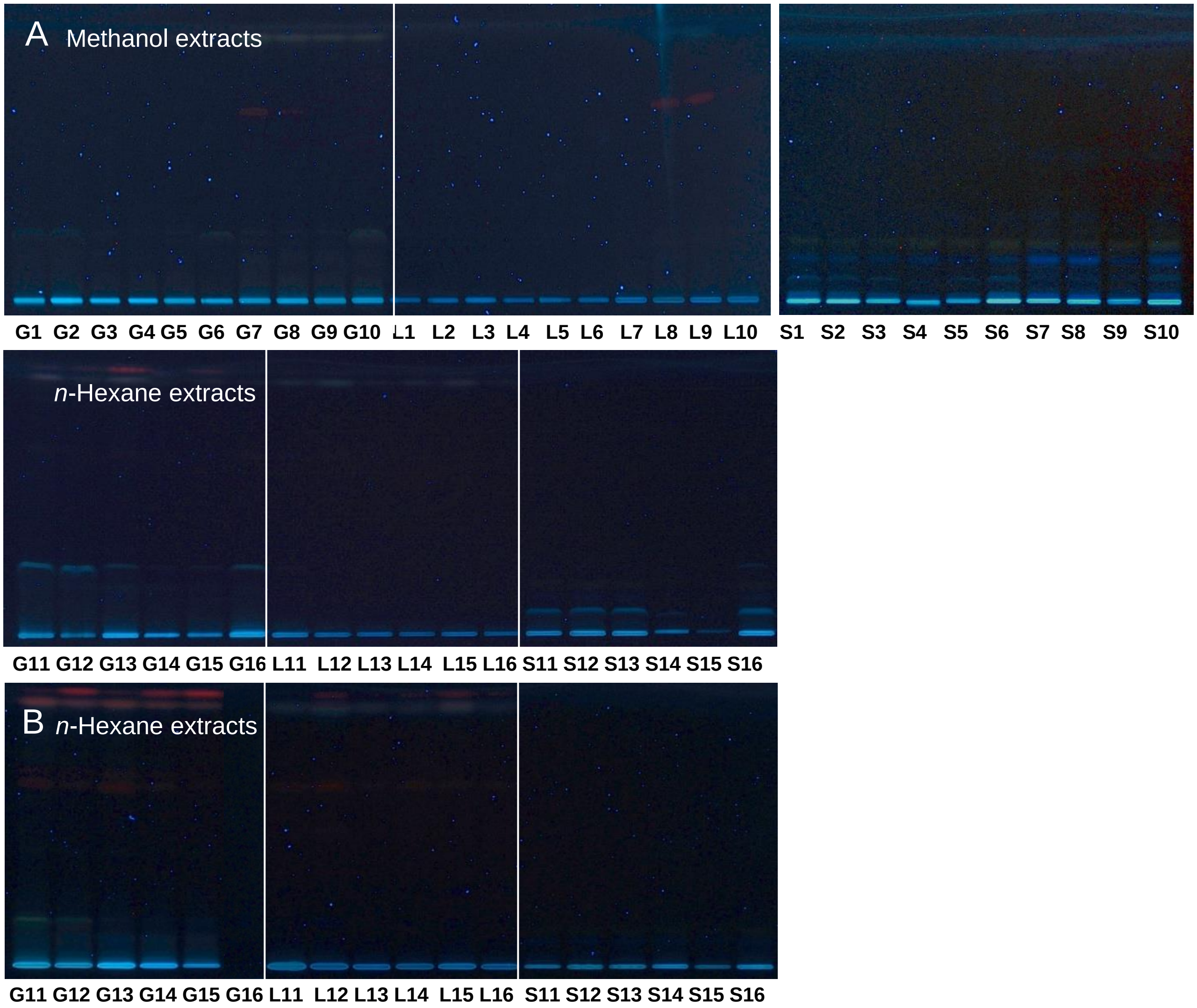

Figure S7. Chromatograms at FLD 366 nm before the *A. fischeri* (A) and *B. subtilis* bioassays (B)

Chromatograms of the three different substrate extracts developed on HPTLC plates silica gel 60 with ethyl acetate – methanol – water 15:3:1 (*V*/*V*/*V*) and detected at FLD 366 nm before the bioassay performance, Related to Figures 3 and 4.

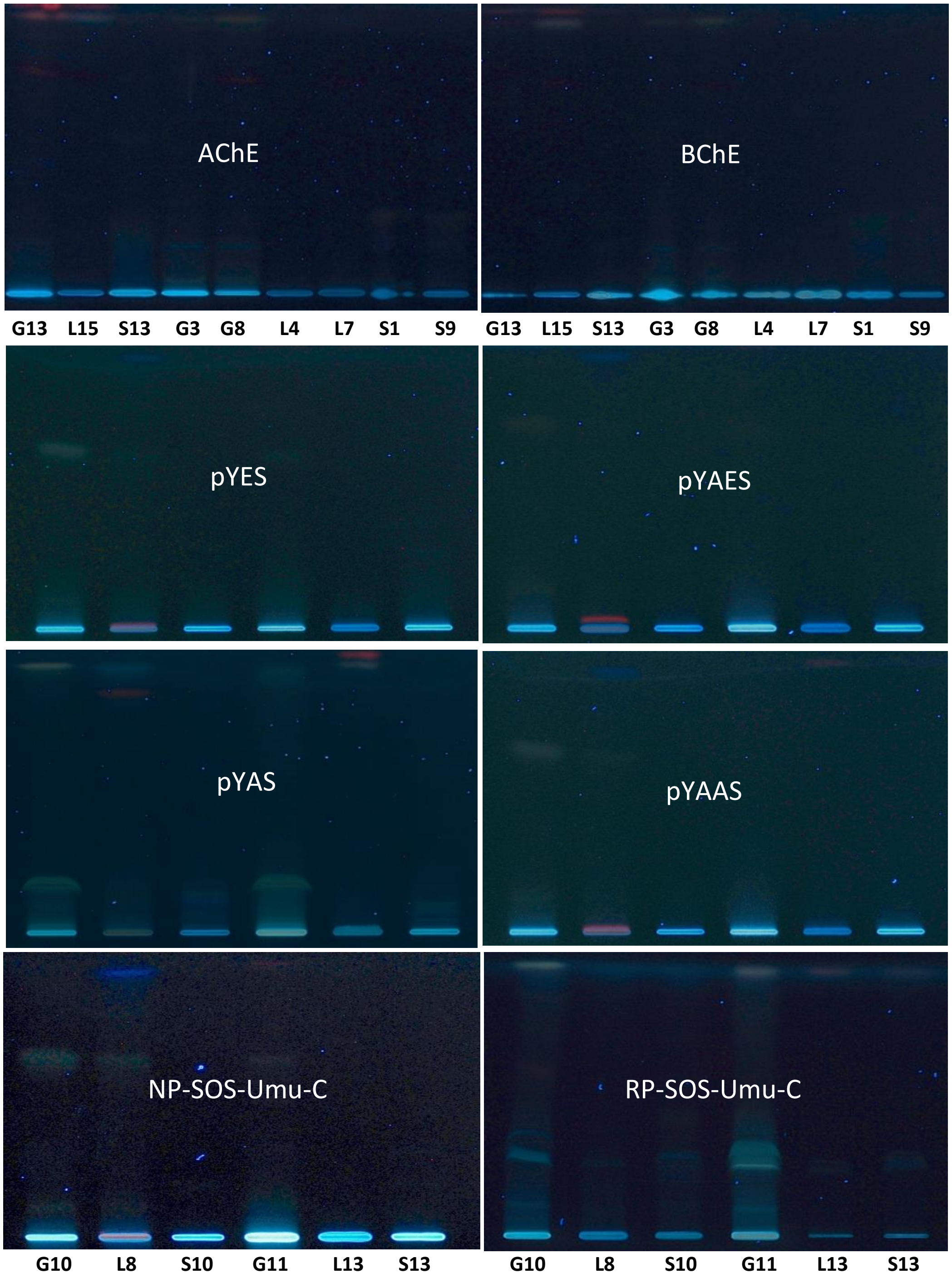

Figure S8. Chromatograms at FLD 366 nm before application of the respective assays

Separation of the *n*-hexane and methanol boat extracts of the three different substrates; respective UV 254 nm chromatograms did not show additional compounds and Vis chromatograms showed orange chlorophylls near the front only (both not depicted), Related to Figures 5 and S9.

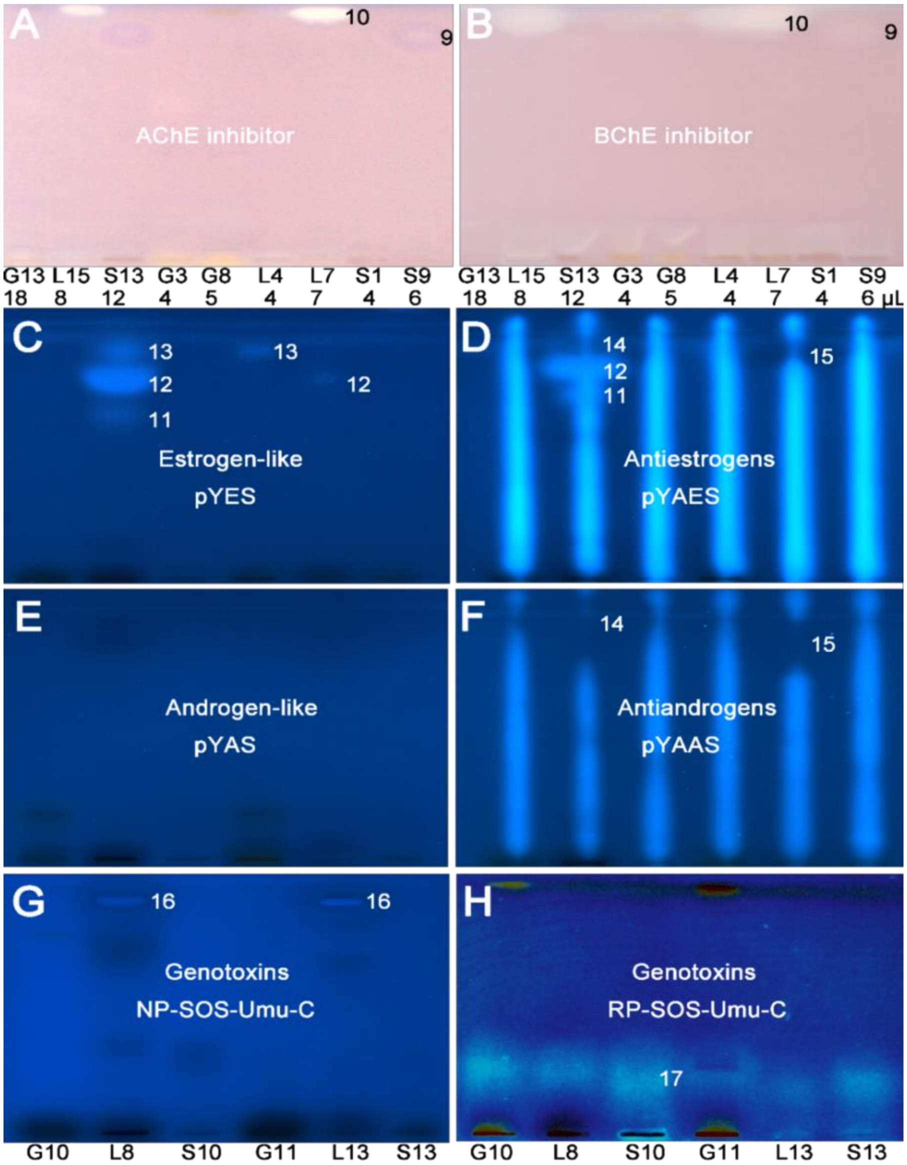

Figure S9. AChE/BChE inhibiting, hormonal and genotoxic compounds

(A) AChE and (B) BChE inhibiting compound zones **9** and **10** evident as colourless/white bands (L7/15 and L4/7, respectively; zone 9 with halo-effect), (C/D) estrogenic (**11**–**13**), (D) antiestrogenic (**14**/**15**), (E/F) androgenic (none), (F) antiandrogenic (**14**/**15**), and (G/H) genotoxic (**16**/**17**) compound zones, evident as (C–H) blue fluorescence or (D/F) fluorescence reduction in the *n*-hexane and methanol boat extracts of the three different substrates (C–H 10 µL/band each as listed at bottom; up to 10 µg/band) developed on HPTLC plates silica gel 60 (except for **H** on RP-18 W plates) with A/B/H ethyl acetate – methanol – water 15:3:1 (*V*/*V*/*V*), C–F *n*-hexane – ethyl acetate 3:4 (*V*/*V*) and G 1:4 (*V*/*V*), detected at A/B white light illumination and C–H FLD 366 nm (respective pre-assay FLD 366 nm chromatograms), Related to Figures 5 and S8.

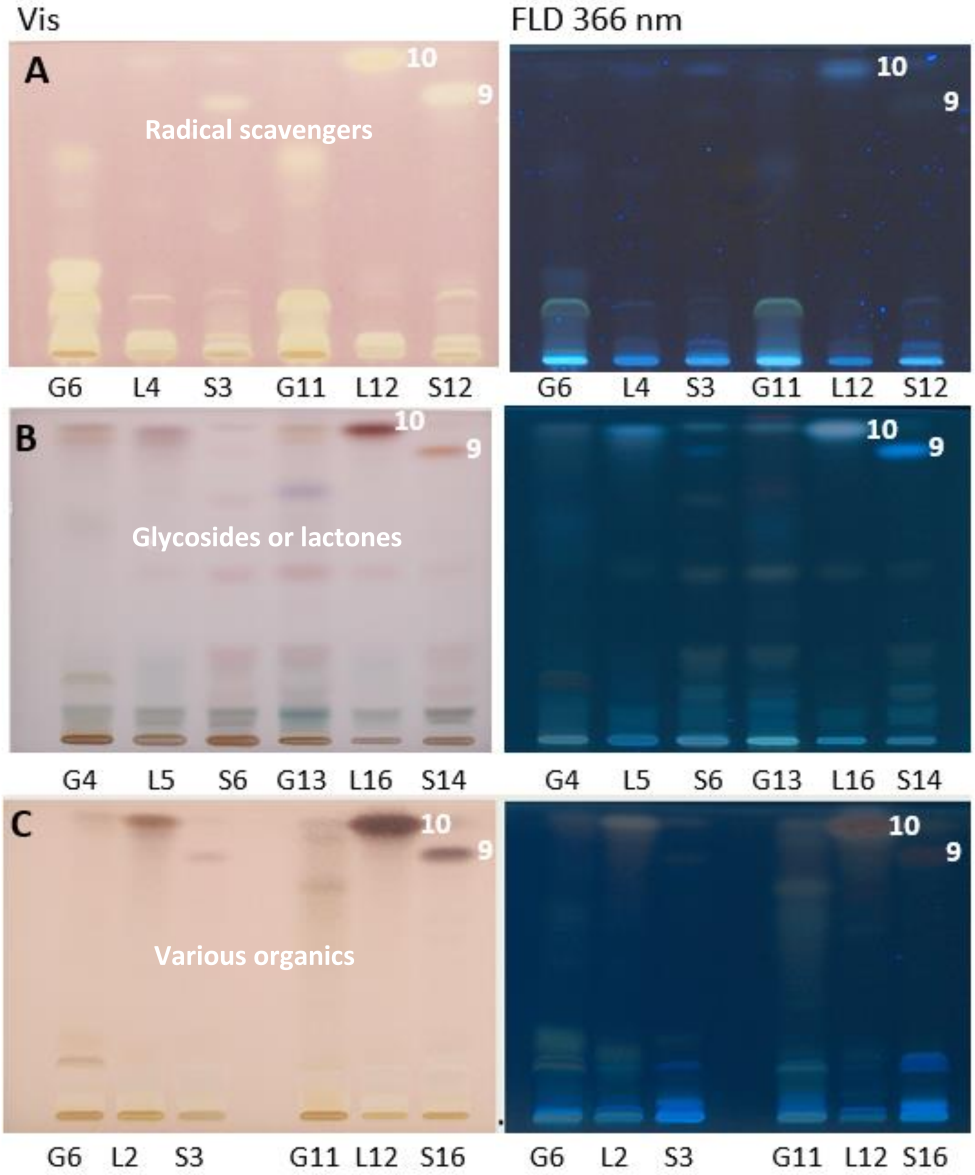

Figure S10. Radical scavenging assay and physico-chemical detection of the chromatograms including derivatization

Chromatograms of the methanol and *n*-hexane extracts of the three different substrates (10 µL each) developed on HPTLC plates silica gel 60 with ethyl acetate – methanol – water 15:3:1 (*V*/*V*/*V*) and detected mainly the more apolar bioactive zones **9** and **10** at Vis and FLD 366 nm after the (A) 2,2-diphenyl-1-picrylhydrazyl (DPPH•) assay for detection of radical scavengers (antioxidants), as well as after derivatization with (B) 2% diethylamine aniline sulphuric acid reagent for detection of glycosides or lactones; (saccharides are supposed to be in the start region for the given mobile phase); (C) 1% vanillin sulfuric acid reagent for more universal detection of organic compounds like sequiterpene derivatives. Related to Figure 2.

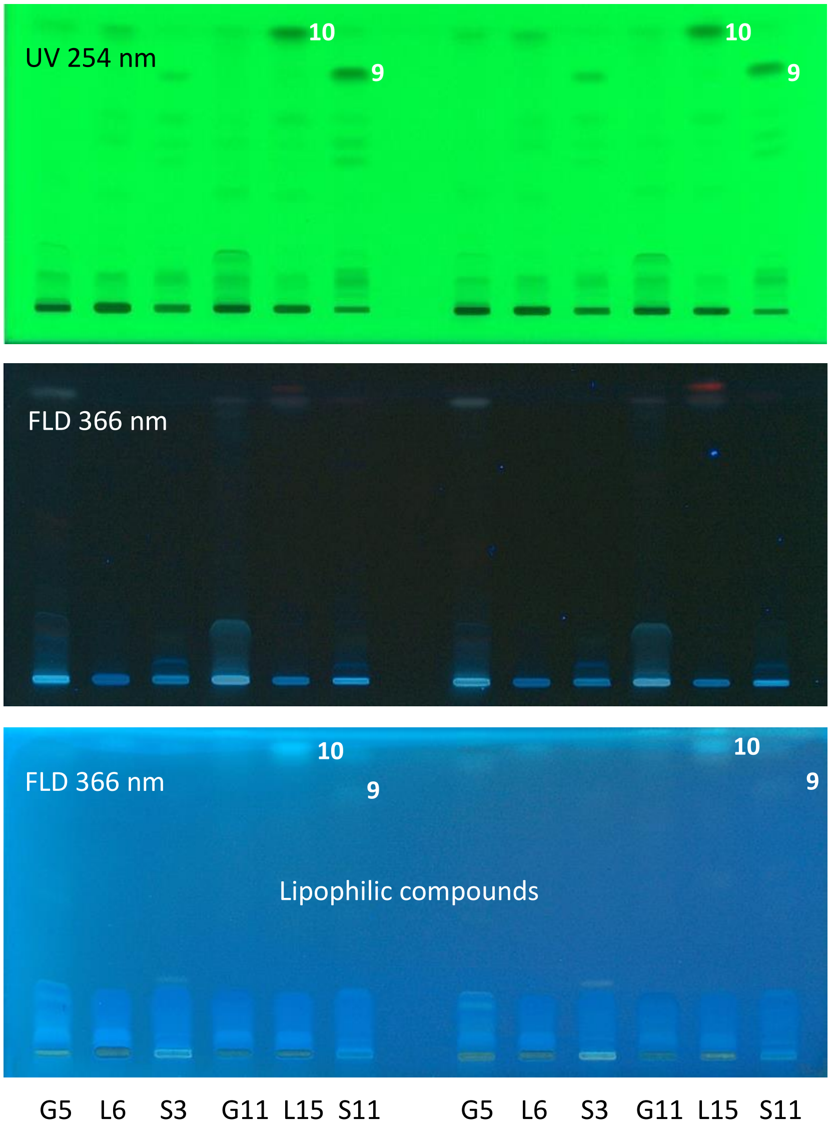

Figure S11 Physico-chemical detection of the chromatograms after primuline reagent

Chromatograms of the methanol and *n*-hexane extracts of the three different substrates (10 µL each) developed on HPTLC plates silica gel 60 with ethyl acetate – methanol – water 15:3:1 (*V*/*V*/*V*) and detected at UV 254 nm (native UV-absorbance of the bioactive zones **9** and **10**) and at FLD 366 nm before and after physisorption of the primuline reagent for detection of lipophilic compounds as blue fluorescent bands. Related to Figure 2.

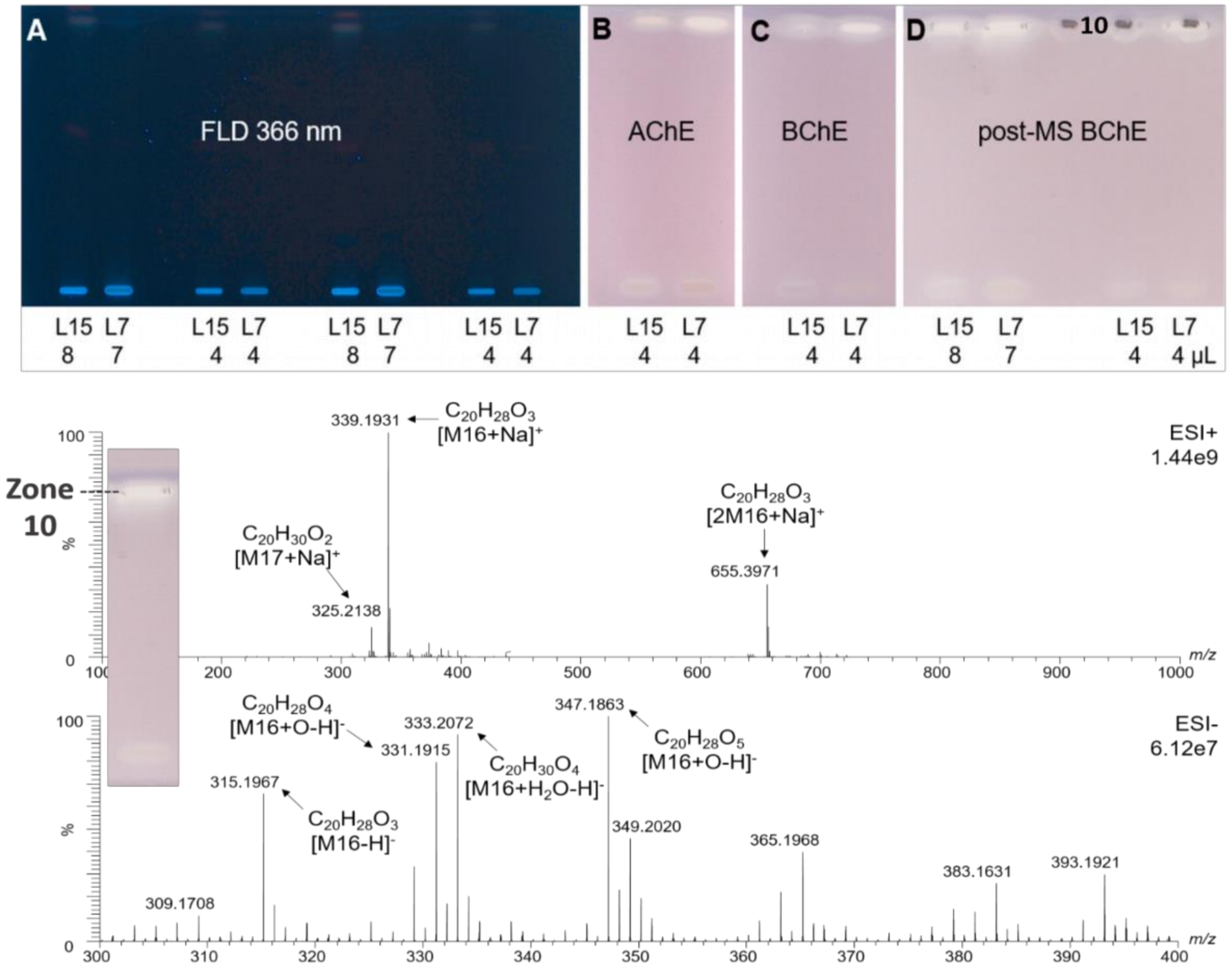

Figure S12. Proof of proper positioning and mass spectra recorded for zone 10

(A) FLD 366 nm chromatogram developed on HPTLC plates silica gel 60 with ethyl acetate – methanol – water 15:3:1 (*V*/*V*/*V*) each of a methanol boat (L15) and *n*-hexane extract (L7) of the leather coral, (B) respective acetyl- and butyrylcholinesterase (AChE/BChE) inhibition autogram, and (C) plate duplicate used for the recording of mass spectra followed by post-MS BChE assay application to prove the proper positioning of the elution head on the zones (fully automated operation in Video S7). (D) Mass signal assignment of zone **10**. Related to Figure 3.

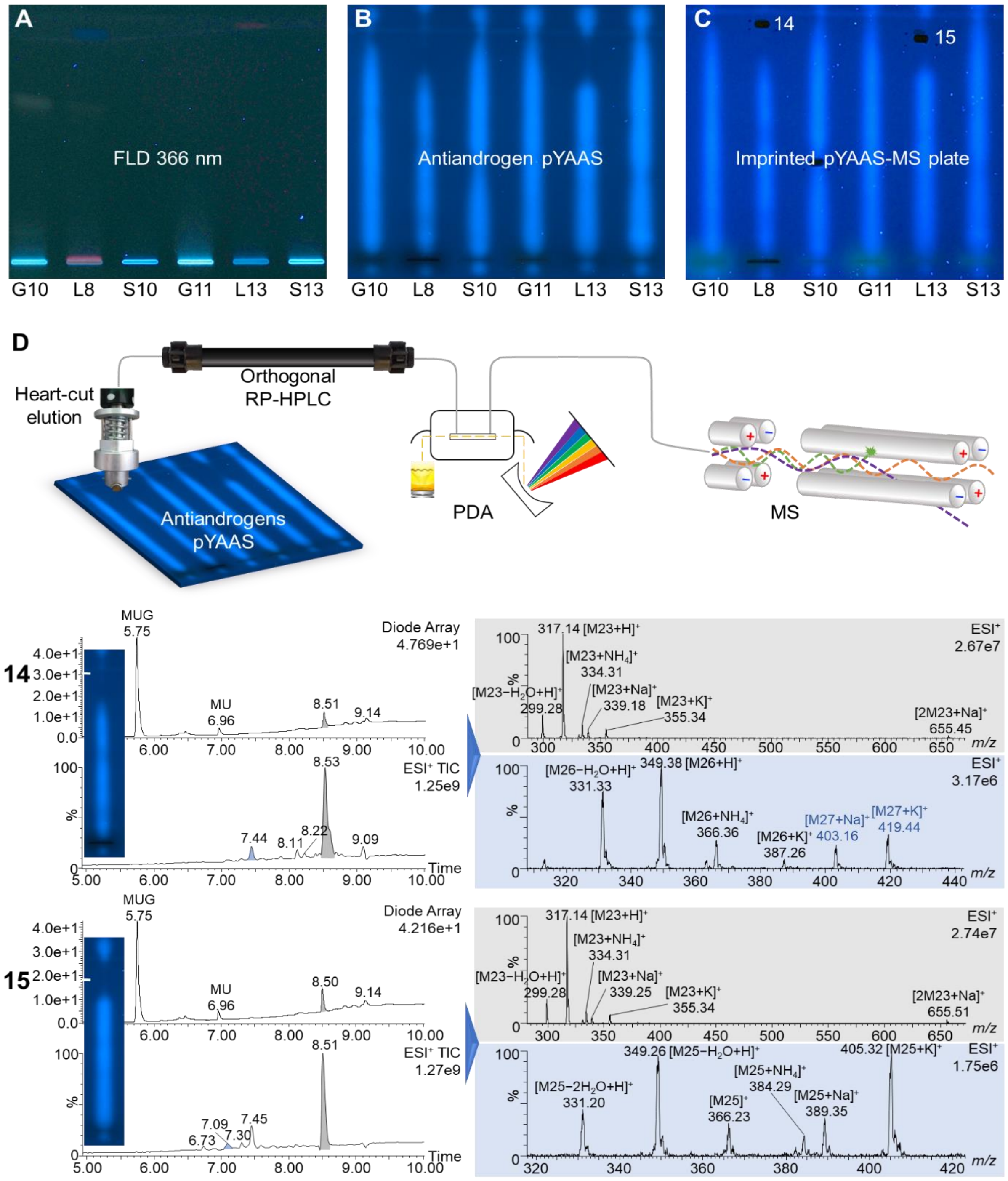

Figure S13. Characterization of the antiandrogenic zones 14 and 15 directly from the pYAAS bioautogram

(A) Chromatogram at FLD 366 nm on HPTLC plate silica gel 60 with ethyl acetate – methanol – water 15:3:1 (*V*/*V*/*V*) of the methanol boat and *n*-hexane extracts of the three distinct substrates, (B) respective pYAAS bioautogram and (C) elution head imprint, verifying proper positioning of the elution head on the zones. (D) Schematic overview of the zone characterization by NP-HPTLC−pYAAS bioassay−RP-HPLC−DAD−HESI-MS and recorded PDA or MS-TIC chromatograms as well as extracted mass spectra (same colour) with assigned mass signals. Related to Figures 5 and S9.
